# Humanitarian Food Security Interventions during the COVID-19 Pandemic in Low- and Middle-Income Countries: A Review of Actions among Non-State Actors

**DOI:** 10.3390/nu13072333

**Published:** 2021-07-08

**Authors:** Warren Dodd, Amy Kipp, Monica Bustos, Aliya McNeil, Matthew Little, Lincoln Leehang Lau

**Affiliations:** 1 School of Public Health Sciences, University of Waterloo, Waterloo, ON N2L 3G1, Canada; amyhkipp@gmail.com (A.K.); mcbustos@uwaterloo.ca (M.B.); am4mcneil@uwaterloo.ca (A.M.); lincoln.lau@caremin.com (L.L.L.); 2 School of Public Health and Social Policy, University of Victoria, Victoria, BC V8W 2Y2, Canada; matthewlittle@uvic.ca; 3 International Care Ministries, Manila 1605, Philippines; 4 Dalla Lana School of Public Health, University of Toronto, Toronto, ON M5T 3M7, Canada

**Keywords:** food access, food availability, hunger, non-governmental organizations, United Nations, COVID-19 pandemic

## Abstract

Widespread food insecurity has emerged as a global humanitarian crisis during the coronavirus disease 2019 (COVID-19) pandemic. In response, international non-governmental organizations (INGOs) and United Nations (UN) agencies have mobilized to address the food security needs among different populations. The objective of this review was to identify and describe food security interventions implemented by INGOs and UN agencies during the early stages of the pandemic. Using a rapid review methodology, we reviewed food security interventions implemented by five INGOs and three UN agencies between 31 December 2019 and 31 May 2020. Descriptive statistical and content analyses were used to explore the extent, range, and nature of these interventions. In total, 416 interventions were identified across 107 low- and middle-income countries. Non-state actors have developed new interventions to directly respond to the food security needs created by the pandemic. In addition, these humanitarian organizations have adapted (e.g., new public health protocols, use of technology) and reframed existing initiatives to position their efforts in the context of the pandemic. These findings provide a useful baseline to monitor how non-state actors, in addition to the food security interventions these organizations implement, continue to be influenced by the pandemic. In addition, these findings provide insights into the different ways in which INGOs and UN agencies mobilized resources during the early and uncertain stages of the pandemic.

## 1. Introduction

In the context of the novel coronavirus disease 2019 (COVID-19), food insecurity has emerged as a global humanitarian crisis. The Executive Director of the United Nations (UN) World Food Programme (WFP) has warned that the world is facing a “hunger pandemic” [1], with estimates suggesting that the number of individuals suffering from acute hunger could double by the end of 2020, affecting 265 million people worldwide [2]. This crisis is particularly pressing in low- and middle-income countries (LMICs), where food insecurity was already a serious concern prior to the COVID-19 pandemic [3,4,5,6]. Across LMICs, food security challenges are disproportionately felt by vulnerable populations, such as children, women, older adults, informal workers, and migrants and refugees, who may not have the financial or social capital to buffer against the shocks created by the pandemic [7,8,9].

Control measures to limit the spread of COVID-19 have impacted all aspects of food systems, including the production, distribution, and storage of food, as well as food environments, consumption, and waste [4,7,9]. For example, in the early stages of the pandemic, food availability was restricted due to trade and mobility restrictions, transportation disruptions, interruptions to agricultural practices, rising demand for food, and an increase in food prices as a result of panic buying and temporary food shortages [4,7,10]. Access to food has also been limited, as government measures restricting mobility have led to reduced employment in both formal and informal sectors, in addition to the decreased ability of vulnerable populations to purchase food or access food through their previous channels, such as daily markets, street vendors, or school-based food programs [5,6,11,12]. Furthermore, the ability to adequately utilize food has been challenged, as households adapt to pandemic measures by purchasing shelf-stable foods or by relying on emergency food rations, with fresh and nutritious fruits and vegetables becoming less available [4,13]. Research from the first several months of the pandemic estimated that the interruption of existing nutrition interventions could drastically heighten instances of childhood and maternal malnutrition [13,14,15]. In this way, the pandemic has further exacerbated the instability of food security in many LMICs, compounding challenges associated with poverty, conflict, and environmental crises, with the potential to lead to long-term malnutrition and other negative health outcomes [4,12].

Non-state actors, such as international non-governmental organizations (INGOs) and UN agencies, play a key role in meeting the needs of vulnerable populations during humanitarian crises [16,17,18]. To respond to the challenges arising from the COVID-19 pandemic, non-state actors have enhanced their response to the ongoing and emerging needs of vulnerable populations, including through interventions aimed at food production and consumption [11,19]. According to the UN’s Global Humanitarian Response Plan: COVID-19, NGOs and UN agencies have played an important role in the implementation of these responses by both expanding their reach and maintaining existing interventions [19]. The role of the humanitarian sector in distributing food, productive agricultural inputs, and cash transfers, as well as providing technical assistance was highlighted in the UN’s plan as crucial to sustaining food production and maintaining the purchasing power of vulnerable groups throughout the pandemic [19].

The research question guiding this review was: How have key humanitarian INGOs and UN agencies addressed food insecurity in LMICs during the early stages of the COVID-19 pandemic? To address this research question, and using a rapid review methodology, this study had two objectives: (1) to identify the number and geographic distribution of food security interventions implemented by key INGOs and UN agencies in the context of the COVID-19 pandemic, and (2) to explore the nature of these interventions, including the types of interventions implemented, the rationale for the interventions, and the partnerships and strategies involved in implementation. 

The early stages of the pandemic were marked by uncertainty and the rapid mobilization of resources to address the negative socioeconomic consequences of the pandemic. By reviewing interventions during the early stages of the pandemic, we aimed to identify and describe the initial initiatives and efforts by select humanitarian non-state actors to better understand how this sector initially reacted and responded to this time of uncertainty and rapid resource mobilization.

## 2. Materials and Methods

We conducted a rapid review of documents published by key INGOs and UN agencies concerning their food security interventions administered in the context of the COVID-19 pandemic. A rapid review methodology was appropriate, as these reviews have the goal of providing timely and systematized research on rapidly evolving situations, such as the COVID-19 pandemic [20,21]. To meet this goal, this review included targeted research objectives, a systematic search approach, and a focus on key non-state actors addressing food insecurity in LMICs between 31 December 2019 and 31 May 2020. 

### 2.1. Non-State Actors Included in Review

INGOs were selected based on the Government of Canada’s (2019) list of major organizations providing international emergency aid [22], which includes: CARE (Cooperative for Assistance and Relief Everywhere) Canada, the International Committee of the Red Cross (ICRC), the International Federation of Red Cross and Red Crescent Societies (IFRC), Médecins Sans Frontières (MSF), Oxfam International, and World Vision International. This list was expanded to include each INGO’s international-, sub-, and country-level offices (e.g., CARE Canada was expanded to include CARE International, CARE Members and Affiliates, such as CARE United Kingdom, and CARE country offices). UN agencies were selected based on the UN’s list of agencies delivering humanitarian assistance [18], which includes: the United Nations Development Programme (UNDP), the United Nations Refugee Agency (UNHCR), the United Nations Children’s Fund (UNICEF) and the World Food Programme (WFP). Furthermore, to be included, organizations must have administered interventions addressing some aspect of food security in the context of the COVID-19 pandemic and provided specific details of the interventions taking place at the country level. Based on these requirements, MSF and UNDP were excluded from our sample. Although three MSF interventions were identified as having some aspect that addressed food insecurity, MSF was excluded from our final analysis because few details were provided of each intervention, making it difficult to incorporate MSF into the analysis. Additionally, UNDP was excluded from the review because the agency’s report outlining their COVID-19 pandemic response, COVID-19—UNDP’s Integrated Response, did not mention food security or related humanitarian interventions (e.g., nutrition, agriculture, food assistance, etc.) (see Table 1). 

### 2.2. Search Strategy and Inclusion Criteria

A systematic search strategy was used to explore both INGOs’ and UN agencies’ websites to identify relevant interventions. For INGOs, we first determined the structure of each organization, which differed slightly across INGOs; however, all INGOs had a single international office, with several sub-offices and/or country-level offices tasked with implementing programs. After determining the structure of each INGO, we compiled a list of the countries in which the INGO operated. Based on this list, international, sub, and country office websites were searched for interventions that met the a priori selection criteria.

Interventions were found in: (1) a centralized place on the INGO website listing examples of projects in different countries; (2) the news or blog section of the INGO website, with stories highlighting specific interventions; (3) in regional reports or reports outlining an INGOs’ COVID-19 pandemic response; and (4) by using a targeted search of keywords on each website. Additionally, CARE, World Vision, and Oxfam interventions were also identified by navigating to the “Where We Work” sections of their international websites and following country-specific links. Finally, a Google search was conducted for each organization, to identify any missed interventions, using the INGO’s name and keywords (see Table S1 for a detailed search strategy for each organization).

To search the websites of UN agencies, we first looked for interventions on each website’s COVID-19 pandemic response page. Then, related news stories, blog posts, situation reports, and publications were reviewed. Each country’s profile in the “Where We Work” section of the website was also explored, which led to additional country-specific situation reports. Finally, a targeted search using keywords was conducted on each agency’s website (see Table S1 for a detailed search strategy for each organization).

To be included, interventions had to meet specific criteria based on their content, geographic scope, and reporting time period (see Table 2 for criteria). Each organization was searched by two independent reviewers to ensure all relevant interventions were identified.

### 2.3. Data Entry and Analysis

Once identified, each intervention was recorded in an Excel database. The database was used to collect and categorize specific details about each intervention (see Table S2). For all categories, with the exception of the pillar(s) of food security addressed by the intervention, details were only recorded if explicitly stated by the organization. Categories in which specific details were not provided, such as no details being available on the beneficiaries or scale of an intervention, were recorded as “undefined”. To categorize the pillar(s) of food security addressed by an intervention, we used the Food and Agriculture Organization (FAO) (2008) and the Food Climate Research Network’s (2020) definitions of food security, and categorized interventions accordingly [23,24] (see Table S2).

Descriptive statistics were calculated to interpret and summarize quantitative findings using two rounds of analysis. For both rounds, our unit of analysis was “intervention”, regardless of the reach (i.e., number of beneficiaries) or size (i.e., budget) of the intervention. This unit of analysis was used because information pertaining to intervention reach or size was reported inconsistently or not reported at all, making it difficult to compare these factors across interventions. In the first round of analysis, all identified interventions were included. The second round of analysis included a subset of interventions that contained data (i.e., not “undefined”) for at least two of the following categories: intended beneficiaries; the aspect(s) of the COVID-19 pandemic addressed by the intervention; and local partners involved in implementing the intervention. Descriptive statistics were complemented by a qualitative content analysis that was used to analyze the description and implementation details of each intervention, using a constant comparative approach [25,26]. This qualitative analysis allowed for the identification and exploration of emergent themes and provided useful context to support the quantitative findings.

## 3. Results

### 3.1. Extent and Range of Interventions

**Figure 1:**
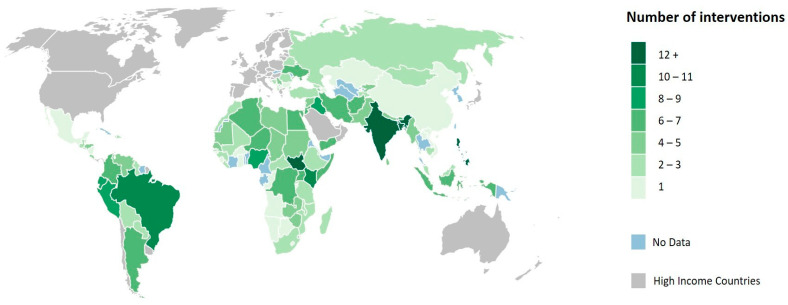
Map showing the geographic distribution of food security interventions implemented in low- and middle-income countries by select INGOs and UN agencies in the context of COVID-19.

We identified 416 food security interventions implemented across 107 LMICs. Most interventions were implemented in Africa (*n* = 155; 37.3%) and Asia (*n* = 153; 36.8%) (see Figure 1). At a country level, the greatest number of interventions were implemented in India (*n* = 19; 4.6%), South Sudan (*n* = 17; 4.1%), and the Philippines (*n* = 16; 3.9%), with an average of 3.89 interventions per country (standard deviation = 3.43). Notably, the distribution of interventions across continents differed between INGOs and UN agencies. For example, UN agencies implemented a higher proportion of interventions in Africa (*n* = 112; 26.9%) compared to INGOs (*n* = 43; 10.3%).

Overall, WFP implemented the most interventions (*n* = 111; 26.7%), followed by IFRC (*n* = 82; 19.7%), and UNHCR (*n* = 66; 15.9%) (see Table 3). To understand the nature of these interventions, we focused on a subset of interventions for which a greater level of detail was available. This subset included 287 interventions across 93 countries.

### 3.2. Nature of Interventions

#### 3.2.1. Intervention Type

The subset of interventions with more complete information included 287 interventions across 93 countries. Implementation most commonly occurred regionally within countries (e.g., the dry corridor in Honduras, the Visayas in the Philippines, etc.; *n* = 87; 30.3%), followed by the national level (*n* = 39; 13.6%) and the community level (*n* = 55; 19.2%). This trend was observed across all organizations (see Table 4). In total, 11 distinct types of interventions were documented. The distribution of food aid (*n* = 171; 59.6%) was the most frequently implemented type of intervention and involved the distribution of dry food rations, basic food baskets, and fruits and vegetables. Other common interventions included providing cash transfers (*n* = 62; 21.6%); implementing nutrition (e.g., micronutrient supplements) (*n* = 36; 12.5%) and feeding programs (e.g., providing cooked meals) (*n* = 27; 9.4%); monitoring, technical, and policy support (*n* = 24; 8.4%); and supporting local food production (*n* = 21; 7.3%) (e.g., distributing seeds). Additionally, interventions often addressed food security in multiple ways through a single intervention. For example, a UNHCR intervention in Syria provided food baskets, hot meals, and multi-purpose cash grants [27]. Similarly, in the Philippines, a CARE intervention provided vulnerable families with rice while also working directly with small local producers to purchase and distribute baskets of fresh vegetables to urban populations [28].

Differences existed between the types of interventions implemented by INGOs and UN agencies. Specifically, a greater proportion of INGO interventions involved food aid distribution (*n* = 103; 74.6% of INGO interventions) compared to interventions delivered by UN agencies (*n* = 68; 45.6% of UN agency interventions). Additionally, 14.1% (*n* = 21) of UN interventions focused on monitoring, technical support, and policy, whereas only 2.2% (*n* = 3) of INGO interventions included these components. Nutrition interventions were also more common among UN agencies (*n* = 27; 18.1% of all UN agency interventions) in comparison to INGOs (*n* = 9; 6.52% of INGO interventions), with the largest proportion of nutrition programs implemented by UNICEF (*n* = 18; 12.1% of UN agency interventions; 50.0% of UNICEF interventions). 

Although most interventions focused on providing immediate humanitarian aid and addressing acute food insecurity, both INGOs and UN agencies also implemented programs focused on addressing broader food system issues, such as supporting local food production and the food supply chain. For example, with reference to the distribution of dry food rations to agricultural and migrant workers in India, Oxfam described their humanitarian approach as being three-phased and incorporating immediate (e.g., dry food rations), intermediate (e.g., unconditional cash transfer), and long-term interventions (e.g., employment opportunities) [29]. In Niger, the WFP focused their work on expanding a social protection program in an effort to address chronic vulnerability, in addition to the current food security and nutrition crises exacerbated by the COVID-19 pandemic [30].

**Figure 2:**
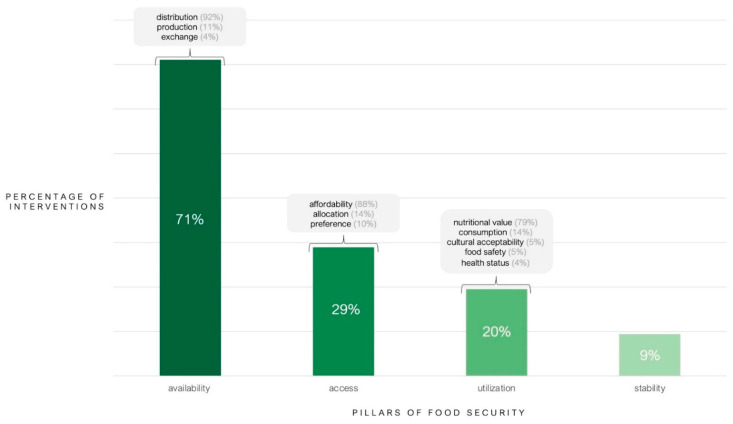
Pillar of food security addressed by interventions delivered by INGOs and UN agencies in response to the COVID-19 pandemic in low- and middle-income countries from 31 December 2019 to 31 May 2020 (*n* = 287). Each category was not mutually exclusive. Thus, interventions could have incorporated more than one pillar of food security. Within each pillar of food security (i.e., availability, access, utilization, and stability), the various components comprising the pillar were calculated as proportions of the number of interventions in the corresponding pillar. For example, approximately 92% of all food availability interventions included food distribution.

With respect to the four pillars of food security (i.e., availability, accessibility, utilization, and stability), the majority of interventions addressed food availability (*n* = 204; 71.1%), with 92.2% (*n* = 188) of these interventions focused on activities such as delivering emergency food aid or adapting existing feeding programs. Of note, 86.2% (*n* = 119) of INGO interventions addressed food availability, compared to 57.0% (*n* = 85) of UN agency interventions. Enhancing food access was the next most frequently addressed pillar of food security (*n*= 83; 28.92% of all interventions), with a greater proportion of UN agency interventions including a food access component (*n* = 52; 34.9% of UN agency interventions) compared to INGO interventions (*n* = 31; 22.5% of INGO interventions). Interventions with a food access component commonly addressed food affordability (*n* = 73; 88.0% of all food access interventions) through cash-based transfers or food vouchers. Additionally, 19.5% (*n* = 56) of all interventions addressed food utilization, with 78.6% (*n* = 44) of these interventions focused on nutrition, through the provision of micronutrient supplements or nutrition initiatives that included the distribution of fruits and vegetables. Food stability was addressed by 9.4% (*n* = 27) of all interventions, with 66.7% (*n* = 18) of these interventions focused on supporting a stable supply of food through maintaining livelihoods for subsistence farmers, ensuring grocery stores remained open, procuring food for local governments, and monitoring and adjusting programs to adapt to changing food prices (see Figure 2).

Interventions were frequently delivered in combination with other forms of aid, such as hygiene supplies, medication, personal protective equipment (PPE), and educational materials. In South Africa, for example, UNHCR provided refugees and asylum-seekers with food parcels, cash assistance, legal assistance, and support for students to continue their education during the pandemic [31]. All organizations provided education specifically related to COVID-19, which was a frequent addition to many food security interventions. For example, a World Vision intervention in Brazil partnered with local parishes to distribute food and sanitation packages, as well as COVID-19 education [32].

#### 3.2.2. Response

Food security interventions most frequently responded to the impacts of government measures used to restrict movement (*n* = 118; 41.1%), such as curfews or city-wide lockdowns. For example, in Sri Lanka, where an island-wide curfew was in effect, World Vision Sri Lanka implemented an intervention delivering food aid to individuals who were unable to engage in their livelihoods because of government restrictions [33]. Although government measures to restrict movement were the most frequent concern that both INGOs and UN agencies addressed, a greater proportion of INGO interventions explicitly responded to these measures (*n* = 78; 56.5% of INGO interventions), compared to interventions delivered by UN agencies (*n* = 40; 26.8% of UN agency interventions) (see Table 5). Conversely, a greater proportion of interventions delivered by UN agencies responded to school and feeding program closures (*n* = 29; 19.5% of UN agency interventions) compared to INGO interventions (*n* = 13; 9.4% of INGO interventions). WFP implemented the most interventions responding to school feeding program closures (*n* = 22; 52.4% of all interventions focused on school feeding program closures; 31.4% of WFP interventions), with many existing programs being adapted from school-based programs to take-home food rations for students.

Interventions were primarily intended to target families (*n* = 80; 27.9%); infants, children, and young people (*n* = 66; 23.0%); and refugees and internally displaced people (IDPs) (*n* = 63; 22.0%). Among the interventions intended for families (*n* = 80), most interventions were designed to respond to government measures that restricted mobility (*n* = 38; 47.5%) or unemployment/reduced cash flow (*n* = 28; 35.0%). Of the interventions intended for infants, children, and young people (*n* = 66), WFP (*n* = 23; 34.9%), UNICEF (*n* = 19; 28.8%), and World Vision (*n* = 15; 22.7%) most commonly led interventions that targeted this group. Additionally, interventions targeting infants, children, and young people were closely linked with the closure of school-based food programs (*n* = 32; 48.5% of interventions targeting this group). Interventions intended for refugees and IDPs (*n* = 63) were most frequently implemented by UNHCR (*n* = 30; 47.6%); however, all organizations provided interventions for this population. 

#### 3.2.3. Partnerships and Implementation

Implementing interventions frequently included collaboration between non-state actors and other sectors or partners. For example, to distribute food baskets to vulnerable families in Iraq, Oxfam worked with local partners across the country, including local authorities, communities, small businesses, and entrepreneurs [34]. A greater proportion of interventions delivered by UN agencies partnered with national governments (*n* = 70; 47.0% of UN agency interventions) and other UN agencies (*n* = 32; 21.5% of UN agency interventions) in comparison with INGO interventions (*n* = 35; 25.4% and *n* = 10; 7.2% of INGO interventions, respectively). Of the interventions that partnered with national governments (*n* = 105), most interventions focused on distributing food aid (*n* = 66; 62.9%), providing cash transfers (*n* = 24; 22.9%), and delivering nutrition programs (*n* = 20; 19.1%). Conversely, a greater proportion of INGO interventions partnered with local governments, community volunteers, and business partners. For example, 30.0% (*n* = 15) of World Vision interventions included partnerships with local governments. Of the interventions that were implemented in partnership with community volunteers (*n* = 49) and businesses (*n* = 35), IFRC more frequently collaborated with both of these partners compared with other organizations (*n* = 26; 56.5% and *n* = 14; 30.4% of IFRC interventions, respectively) (see Table 6).

**Figure 3:**
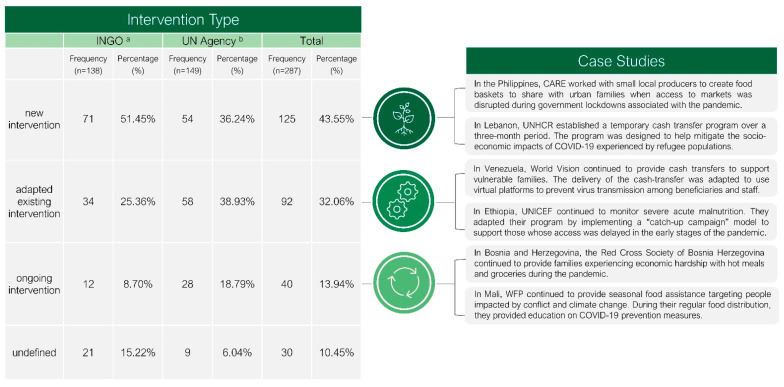
Intervention type and case studies of food security interventions delivered by INGOs and UN agencies in response to the COVID-19 pandemic in low- and middle-income countries from 31 December 2019 to 31 May 2020 (*n* = 287). ^a^ INGO = international non-governmental organization. ^b^ UN Agency = United Nations Agency.

In response to emergent food security challenges associated with the COVID-19 pandemic (e.g., increased unemployment, school closures, quarantine measures, disrupted supply chains, increased costs of goods, etc.), INGOs and UN agencies most commonly developed new interventions (*n* = 125; 43.5%). Of note, a greater proportion of INGO interventions were new (*n* = 71; 51.4% of INGO interventions) compared to interventions delivered by UN agencies (*n* = 54; 36.2% of UN agency interventions). New interventions included a range of activities, from emergency food aid to the delivery of seeds, to cash-based transfers. In contrast to these new interventions, many existing interventions that were in place prior to the pandemic were adapted to meet the changing needs resulting from the pandemic (*n* = 92; 32.1%) (e.g., an increase in populations experiencing food insecurity, limited mobility, decreased accessibility of food, etc.). A greater proportion of interventions delivered by UN agencies were considered “adapted” (*n* = 58; 38.9% of UN agency interventions) compared to INGO interventions (*n* = 35; 25.4% of INGO interventions). In these cases, interventions were often described as “scaled-up”, “extended”, or “revised” (see Figure 3).

Both INGOs and UN agencies relied on existing logistical expertise developed during past emergencies to adapt their interventions. For example, in South Sudan, World Vision’s Ebola Virus Disease Prevention Program was adapted to inform their prevention and control processes in their campaign to address the COVID-19 pandemic [35]. Adaptations were also made to existing implementation processes to ensure that interventions met food security needs while preventing the spread of disease. For example, structural changes were implemented in some cases to ensure physical distancing guidelines could be followed, such as re-arranging and enhancing distribution sites, providing double rations, and delivering food aid directly to the homes of beneficiaries. Additionally, staff and volunteers of organizations were trained in safety measures, including hand-washing and sanitization of supplies, and were provided with proper PPE. For example, in Peru, CARE adapted soup kitchens that provided low-income Peruvians and Venezuelan migrants with take-home meals [36].

Technology was also used to meet the changing needs of populations as well as emerging public health guidelines. Adaptations to existing interventions included the introduction of different forms of technology aimed at reducing in-person contact with beneficiaries, such as online or mobile delivery of cash transfers, telephone monitoring, and online communications. Telephone checks-ins were used by World Vision in Indonesia to monitor and support farmers enrolled in their organic vegetable development program who were affected by drought in addition to the pandemic [37]; in Iran, IFRC conducted COVID-19-related training and brainstorming session via webinars [38]; and in the Democratic Republic of Congo, UNHCR distributed cellphones to IDPs to facilitate mobile cash transfers [39]. Additionally, technology was used to educate beneficiaries on COVID-19 prevention and awareness. In Myanmar, for example, World Vision provided COVID-19 updates via loudspeakers and social media [40]. Similarly, in Bolivia, UNICEF hosted Facebook Live and Zoom webinars to answer questions on nutrition and healthy eating during government measures restricting mobility [41]. 

In addition to adapting existing programs, some interventions continued to operate in the same way as they did prior to the pandemic without adaptations (i.e., ongoing); however, the descriptions of these interventions were reframed to emphasize the importance of the intervention within the context of the pandemic (*n* = 40; 13.94%). A greater proportion of interventions delivered by UN agencies were considered “ongoing” (*n* = 28; 18.8% of UN agency interventions) compared to INGO interventions (*n* = 12; 8.7% of INGO interventions). In Somalia, for example, UNICEF detailed the continuation of existing “essential health and nutrition service provision” as key to maintaining adequate nutrition for women and children during the COVID-19 pandemic [42]. Similarly, in Myanmar, ICRC explained that they would continue the work from the crisis response plan implemented in 2017 during the pandemic, which involved the distribution of food rations to those in need [43].

## 4. Discussion

### 4.1. Multidimensional Vulnerabilities Impacting Food Security

The COVID-19 pandemic has exposed and exacerbated multidimensional vulnerabilities that negatively impact food security in LMICs. The number and geographic breadth of emergency food aid interventions identified in this review highlight the impact of associated economic losses on individual and household food security.

Factors such as national and regional governance (e.g., social protection programs), the existing capacity of non-state actors, and environmental conditions directly impact food security and the ability of LMICs to respond in the context of the COVID-19 pandemic [44,45,46]. In India, for example, where we identified the greatest number of interventions, the nation has experienced stringent lockdown measures and the mass out-migration of internal migrant workers from cities [47,48]. In South Sudan, where we identified the second-highest number of interventions, the threat of food insecurity due to the COVID-19 pandemic exists alongside the challenges of climate change, a fragile healthcare system, and years of internal violence [49]. Indeed, based on the integrated food security phase classification (IPC), over 50% of the population in South Sudan was projected to experience “Crisis” (IPC Phase 3) or worse acute food insecurity during the early stages of the pandemic, due to the aftermath of the flooding in 2019 and low crop production. [50] In the Philippines, where we identified the third-highest number of interventions, the government has enforced aggressive restrictions on movement in many provinces that threaten the ability of populations to access food [51]. Additionally, in many LMICs, the impacts of the COVID-19 pandemic on food security were exacerbated by environmental factors, such as locust swarms in East Africa [52], and flooding caused by weeks of monsoon rains in Bangladesh [53]. In this way, the delivery of humanitarian food assistance is increasingly challenging in the context of other food crisis drivers, such as adhering to public health measures in conflict situations or during extreme weather events.

### 4.2. The Role of Non-State Actors in Addressing Food Security during a Global Pandemic

Our findings highlighted that INGOs and UN agencies played different but complementary roles during the early stages of the COVID-19 pandemic. For example, INGOs appeared to focus more on rapidly providing immediate food aid to vulnerable communities, whereas UN agencies appeared to focus more on supporting food security through high-level interventions such as monitoring, technical support, policy, and nutrition interventions. In addition, these organizations leveraged different types of partnerships to support intervention implementation. Ongoing and enhanced coordination across non-state actors and with international, national, regional, and local governments and stakeholders is key to ensuring that diverse supports for food security—from emergency food aid to technical support—are addressed during the pandemic [54].

Our findings also indicate that in the context of a global pandemic, both INGOs and UN agencies continued to fulfill their organizational mandates, while addressing ongoing and emergent challenges to food security (see Table S1). In many cases, the mandate of the organizations drove the types of interventions that were observed. It is perhaps unsurprising that, in a time of crisis, these actors continue to focus on organizational mandates, as these organizations have expertise in these areas and are often beholden to strategic plans and donor expectations [55]. By relying on their existing expertise, non-state actors can effectively address the various pillars of food security; however, in the context of the COVID-19 pandemic, INGOs and UN agencies must also have the flexibility and the resources required to address the needs of the various populations whose vulnerabilities have been exposed by the pandemic.

In the early stages of the COVID-19 pandemic, humanitarian food security interventions implemented by INGOs and UN agencies have taken many forms, in terms of the types of programs, the aspects of food security addressed, and the partners and practices involved in implementation. Our findings indicated that a large number of interventions were developed in response to the COVID-19 pandemic. The emergence of these new interventions indicates that the pandemic is threatening food security in LMICs in new ways, and that INGOs and UN agencies have responded to address these new challenges. The unprecedented nature of the COVID-19 pandemic necessitates that organizations consider innovative approaches to effectively, and safely, implement their interventions [56]. Examples of innovation among new interventions included the use of new technologies and the creation of new implementation protocols.

Interventions were also categorized as adapted and ongoing, demonstrating that non-state actors were implementing interventions with vulnerable populations in LMICs to address food security challenges prior to the pandemic. To continue to support these populations, non-state actors have had to maintain and adapt existing food security interventions. Among interventions classified as “adapted”, the observed adaptations were in response to both increased food insecurity as well as new public health guidelines, such as physical distancing requirements. Among interventions classified as “ongoing”, our analysis of intervention descriptions demonstrated that organizations have reframed these descriptions to highlight the relevance of interventions in the context of the COVID-19 pandemic.

### 4.3. Limitations

This review has several limitations. First, we have focused on several INGOs and UN agencies identified as key to delivering humanitarian aid; however, this approach limited our ability to assess the work being done by all INGOs or UN agencies delivering food-related humanitarian aid during the early stages of the COVID-19 pandemic. Second, our search strategy only identified interventions that were publicly reported and available via organizational websites and reports. As a result, we may have missed interventions among included INGOs and UN agencies that were not publicly available. Third, the application of inclusion and exclusion criteria during our search process may be biased by the reviewer’s interpretation of these criteria. To mitigate the influence of this bias, two independent reviewers were involved at all stages of the review process, to ensure relevant interventions were identified. Fourth, by including these organizations focused on delivering humanitarian aid, we excluded non-state actors involved in agricultural development and building food system resilience, such as the FAO and the International Fund for Agricultural Development. Finally, we did not synthesize any program evaluations of the interventions included in this review, due to our focus on the early stages of the COVID-19 pandemic. Thus, we are unable to comment on the effectiveness or success of these interventions.

## 5. Conclusions

This review examined how key humanitarian INGOs and UN agencies have responded to food security challenges in LMICs during the early stages of the COVID-19 pandemic. Specifically, we described the extent, range, and nature of food security interventions implemented by these non-state actors across LMICs. We observed that the COVID-19 pandemic has exposed and exacerbated the existing individual, structural, and environmental vulnerabilities that underlie and contribute to food security challenges in many settings. To address these challenges, non-state actors have not only developed new interventions to meet food security needs but have also adapted and reframed existing initiatives to continue to operate during the pandemic.

These findings provide a useful baseline to monitor how non-state actors, in addition to the food security interventions these organizations implement, continue to be influenced by the pandemic. In addition, these findings highlight the different ways in which INGOs and UN agencies mobilized resources during the early and uncertain stages of the pandemic. In many cases, these organizations drew on existing networks, infrastructure, and expertise to inform intervention development and adaptation. Overall, this information is helpful in understanding and informing future responses among non-state actors during subsequent widespread crises where food security is negatively impacted.

## Figures and Tables

**Table 1:** Non-state actors included in the review of interventions addressing food insecurity in the context of COVID-19 in low- and middle-income countries from 31 December 2019 to 31 May 2020 ^a^.

International Non-governmental Organizations	● CARE ^b^
	● International Committee of the Red Cross
	● International Federation of Red Cross and Red Crescent Societies
	● Oxfam
	● World Vision
United Nations Agencies	● United Nations Refugee Agency
	● United Nations Children’s Fund
	● World Food Programme

^a^ INGOs were selected based on the Government of Canada’s (2019) list of major organizations providing international emergency aid. This list was expanded to include each INGO’s international-, sub-, and country-level offices. UN agencies were chosen based on the UN’s (2020) list of agencies delivering humanitarian assistance. ^b^ CARE = Cooperative for Assistance and Relief Everywhere.

**Table 2:** Inclusion and exclusion criteria used to identify interventions addressing food insecurity in the context of COVID-19 in low- and middle-income countries from 31 December 2019 to 31 May 2020.

Inclusion	Exclusion
Intervention addressed food security at any point along the food supply chain	Intervention did not directly address food security
Intervention conducted in a low- and middle-income country	Intervention conducted in a high-income country
Intervention emerged in response to food security challenges brought about by the COVID-19 pandemic, adapted as a result of the pandemic, or described in the context of the pandemic	Intervention not described in the context of the COVID-19 pandemic
Intervention implemented at the country level	Intervention implemented across more than one country
Intervention implemented between 31 December 2019 and 31 May 2020	Intervention implemented before 31 December 2019–31 May 2020

**Table 3:** Frequency and percentage of food security interventions implemented by INGOs and UN agencies in low- and middle-income countries from 31 December 2019 to 31 May 2020 (*n* = 416).

	Total Interventions			Subset of Interventions ^a^	
	Organization	Frequency (*n* = 416)	Percentage (%)	Frequency (*n* = 287)	Percentage (%)
INGO ^b^	IFRC ^c^	82	19.71%	46	16.03%
	World Vision	59	14.18%	50	17.42%
	CARE ^d^	24	5.77%	18	6.27%
	Oxfam	15	3.61%	13	4.53%
	ICRC ^e^	13	3.13%	11	3.83%
UN agency ^f^	WFP ^g^	111	26.68%	70	24.39%
	UNHCR ^h^	66	15.87%	43	14.98%
	UNICEF ^i^	46	11.06%	36	12.54%

^a^ Represents a subset of interventions for which a greater level of detail was available (contained information (i.e., not “undefined”) for at least two of the following categories: intended beneficiaries; the aspect(s) of COVID-19 addressed by the intervention; and local partners involved in implementing the intervention). This subset was the basis for the analysis of the subsequent sections of the results. ^b^ INGO = international non-governmental organization. ^c^ IFRC = International Federation of Red Cross and Red Crescent Societies. ^d^ CARE = Cooperative for Assistance and Relief Everywhere. ^e^ ICRC = International Committee of the Red Cross. ^f^ UN Agency = United Nations Agency. ^g^ WFP = World Food Programme. ^h^ UNHCR = United Nations Refugee Agency. ^i^ UNICEF = United Nations Children’s Fund.

**Table 4:** Characteristics of food security interventions delivered by INGOs and UN agencies in response to the COVID-19 pandemic in low- and middle-income countries from 31 December 2019 to 31 May 2020 (*n* = 287).

	INGO ^a^		UN Agency ^b^		Total	
	Frequency (*n* = 138)	Percentage (%)	Frequency (*n* = 149)	Percentage (%)	Frequency (*n* = 287)	Percentage (%)
Scale						
community	32	23.19%	23	15.44%	55	19.16%
regional	45	32.61%	42	28.19%	87	30.31%
national	18	13.04%	21	14.09%	39	13.59%
undefined	43	31.16%	63	42.28%	106	36.93%
Program type ^c^						
distributing food aid	103	74.64%	68	45.64%	171	59.58%
providing cash transfer	20	14.49%	42	28.19%	62	21.60%
nutrition program	9	6.52%	27	18.12%	36	12.54%
implementing feeding programs	14	10.14%	13	8.72%	27	9.41%
monitoring, technical, and policy support	3	2.17%	21	14.09%	24	8.36%
supporting local food production	12	8.70%	9	6.04%	21	7.32%
supporting food supply chain	4	2.90%	9	6.04%	13	4.53%
providing food voucher	7	5.07%	3	2.01%	10	3.48%
livelihood and income generation	5	3.62%	5	3.36%	10	3.48%
prepositioning and procuring	0	0.00%	5	3.36%	5	1.74%
food safety	0	0.00%	3	2.01%	3	1.05%
other	12	8.70%	3	2.01%	15	5.23%
undefined	1	0.72%	0	0.00%	1	0.35%
Pillar of food security ^c^						
availability ^d^	119	86.23%	85	57.05%	204	71.08%
*distribution*	110	92.44%	78	91.76%	188	92.16%
*production*	12	10.08%	10	11.76%	22	10.78%
*exchange*	1	0.84%	7	8.24%	8	3.92%
access ^d^	31	22.46%	52	34.90%	83	28.92%
*affordability*	27	87.10%	46	88.46%	73	87.95%
*allocation*	4	12.90%	6	11.54%	10	13.70%
*preference*	0	0.00%	1	1.92%	1	10.00%
utilization ^d^	18	13.04%	38	25.50%	56	19.51%
*nutritional value*	12	66.67%	32	84.21%	44	78.57%
*preparation and consumption*	5	27.78%	3	7.89%	8	14.29%
*food safety*	0	0.00%	3	7.89%	3	5.36%
*cultural acceptability*	2	11.11%	1	2.63%	3	5.36%
*health status*	1	5.56%	1	2.63%	2	3.57%
stability ^d^	12	8.70%	15	10.07%	27	9.41%
*stable supply*	7	58.33%	11	73.33%	18	66.67%
*environmental stability*	5	41.67%	4	26.67%	9	33.33%

^a^ INGO = international non-governmental organization. ^b^ UN Agency = United Nations Agency. ^c^ Each category was not mutually exclusive. Thus, interventions could have incorporated more than one component listed. ^d^ Within each pillar of food security (i.e., availability, access, utilization, and stability), the various components comprising the pillar were calculated as proportions of the number of interventions in the corresponding pillar. For example, approximately 92% of all food availability interventions included food distribution.

**Table 5:** Intended beneficiaries of food security interventions delivered by INGOs and UN agencies in response to the COVID-19 pandemic in low- and middle-income countries from 31 December 2019 to 31 May 2020 (*n* = 287).

	INGO ^a^		UN Agency ^b^		Total	
	Frequency (*n* = 138)	Percentage (%)	Frequency (*n* = 149)	Percentage (%)	Frequency (*n* = 287)	Percentage (%)
Aspect of COVID-19 addressed by intervention ^c^						
government measures restricting mobility	78	56.52%	40	26.85%	118	41.11%
unemployment/reduced cash flow	36	26.09%	32	21.48%	68	23.69%
school feeding program closures	13	9.42%	29	19.46%	42	14.63%
disrupted supply chain	10	7.25%	16	10.74%	26	9.06%
environmental vulnerabilities	15	10.87%	10	6.71%	25	8.71%
increased cost of goods	6	4.35%	15	10.07%	21	7.32%
displacement/conflict	4	2.90%	7	4.70%	11	3.83%
existing food insecurity/malnutrition	6	4.35%	3	2.01%	9	3.14%
other	8	5.80%	13	8.72%	21	7.32%
undefined	35	25.36%	38	25.50%	73	25.44%
Intended beneficiaries ^c^						
families	46	33.33%	34	22.82%	80	27.87%
infants/children/young people	23	16.67%	43	28.86%	66	23.00%
refugees/internally displaced people	19	13.77%	44	29.53%	63	21.95%
migrants	20	14.49%	7	4.70%	27	9.41%
women	9	6.52%	13	8.72%	22	7.67%
older adults	13	9.42%	2	1.34%	15	5.23%
farmers	10	7.25%	3	2.01%	13	4.53%
low-income populations	3	2.17%	7	4.70%	10	3.48%
people experiencing homelessness/living in the streets	6	4.35%	3	2.01%	9	3.14%
people with underlying medical conditions	7	5.07%	2	1.34%	9	3.14%
people living with disabilities	7	5.07%	2	1.34%	9	3.14%
rural/remote areas	2	1.45%	4	2.68%	6	2.09%
informal sector workers/daily wage earners	4	2.90%	2	1.34%	6	2.09%
frontline workers—health care workers	4	2.90%	0	0.00%	4	1.39%
frontline workers—volunteers	2	1.45%	2	1.34%	4	1.39%
Indigenous Peoples	3	2.17%	0	0.00%	3	1.05%
other	15	10.87%	15	10.07%	30	10.45%
undefined vulnerable population	12	8.70%	12	8.05%	24	8.36%
undefined	7	5.07%	9	6.04%	16	5.57%

^a^ INGO = international non-governmental organization. ^b^ UN Agency = United Nations Agency. ^c^ Each category was not mutually exclusive. Thus, interventions could have incorporated more than one component listed.

**Table 6:** Partnerships involved in food security interventions implemented by INGOs and UN agencies in response to the COVID-19 pandemic in low- and middle-income countries from 31 December 2019 to 31 May 2020 (*n* = 287) ^a^.

	INGO ^b^		UN Agency ^c^		Total	
	Frequency (*n* = 138)	Percentage (%)	Frequency (*n* = 149)	Percentage (%)	Frequency (*n* = 287)	Percentage (%)
national government	35	25.36%	70	46.98%	105	36.59%
other NGO ^d^	38	27.54%	30	20.13%	68	23.69%
community volunteers	40	28.99%	9	6.04%	49	17.07%
UN agency	10	7.25%	32	21.48%	42	14.63%
business	25	18.12%	10	6.71%	35	12.20%
local government	18	13.04%	6	4.03%	24	8.36%
health workers	11	7.97%	7	4.70%	18	6.27%
regional government	5	3.62%	6	4.03%	11	3.83%
faith-based organization	5	3.62%	1	0.67%	6	2.09%
other	12	8.70%	14	9.40%	26	9.06%
undefined	33	23.91%	37	24.83%	70	24.39%

^a^ Each category was not mutually exclusive. Thus, interventions could have incorporated more than one component listed. ^b^ INGO = international non-governmental organization. ^c^ UN Agency = United Nations Agency. ^d^ NGO = non-governmental organization.

## Data Availability

The full database for this review is included in Table S3.

## Supplementary Materials

The following are available online at https://www.mdpi.com/article/10.3390/nu13072333/s1, Table S1: Details on search strategy for each non-state actor included in the review, Table S2: List of details extracted on food security interventions in the context of the COVID-19 pandemic, Table S3. Characteristics of food security interventions implemented in response to the COVID-19 pandemic by INGOs and UN agencies in low-and-middle income countries from 31 December 2019 to 31 May 2020 (*n* = 287).
