# Supplementary material for: Humanitarian Food Security Interventions during the COVID-19 Pandemic in Low- and Middle-Income Countries: A Review of Actions among Non-State Actors
Source: Nutrients. 2021 Jul 8;13(7):2333. doi: 10.3390/nu13072333 (PMC8308552; doi:10.3390/nu13072333)
Supplement: Supplementary file 1 [file nutrients-13-02333-s001.zip › nutrients-1236356-supplementary.pdf]

SUPPLEMENTARY FILE

Table S1. Details on search strategy for each non-state actor included in the review

CARE

Purpose: CARE works around the globe to save lives, defeat poverty and achieve social justice. CARE places particular emphasis on strengthening the voice of women and girls and enabling them to influence the decisions that affect their lives.

Structure of Organization: CARE International is a global confederation of 14 National Members, 3 Candidates and 1 Affiliate with a common vision and mission to defeat poverty. Each CARE Member is an independent organization that leads programs, raises funds, advocates on key issues, communicates to the public in their country, and supports the work of CARE's programming in 95 countries across the world. CARE USA, CARE Canada, and CARE Australia are Lead Members for specific country offices. Lead Members as well as other CARE International members provide technical assistance and support for programs/projects being implemented in Country Offices where they are engaged.

Source: CARE International. (2020). *CARE.* Retrieved from: https://www.care-international.org/

Search Strategy

1. The list of countries where CARE operates was identified on CARE International's website under the "Where We Work" page. Based on this list of countries, the researcher clicked on each country specific link, which redirected them to the country's profile within the CARE International website. In many instances, this profile provided a link to the specific Country Office website. In a few cases the profile provided a link to a page on a Lead Members' website that highlighted the specific country.

2. Once the Country Office or Lead Members' website was identified the researcher searched for information related to COVID-19 and food security. This information was found:
    a. In one centralized spot highlighting the Country Office or Lead Members' COVID-19 response;
    b. In the news section of the website, with stories highlighting specific interventions addressing COVID-19; and
    b. By using a targeted search for food security interventions in the context of COVID-19 (keywords searched: "COVID" OR "corona" AND "food" OR "seed" OR "nutrition" OR "agriculture" OR "meal" OR "ration")

3. To complement this search, all Member, Candidates and Affiliate websites were also searched with interventions identified in:
    a. In one centralized spot highlighting the Member, Candidate, or Affiliate's COVID-19 response
    b. In the news section of the website, with stories highlighting specific interventions addressing COVID-19
    b. By using a targeted search for food security interventions in the context of COVID-19 (keywords searched: "COVID" OR "corona" AND "food" OR "seed" OR "nutrition" OR "agriculture" OR "meal" OR "ration")

4. As a final complementary step, a targeted Google search was conducted to identify any remaining interventions not captured in the first two search methods. (keywords searched: "CARE" AND "COVID" OR "corona" AND "food" OR "seed" OR "nutrition" OR "agriculture" OR "meal" or "ration")

Oxfam

Purpose: Oxfam is a global movement of people who are fighting inequality to beat poverty together. Oxfam tackles the inequalities that make and keep people poor.

Structure of Organization: Oxfam works in more than 90 countries, with thousands of partners, allies, and communities. Oxfam is composed of 20 member organizations, called Affiliates, who coordinate and lead programs via the Oxfam International Secretariat. The International Secretariat facilitates and supports collaboration between Oxfam's Affiliates to provide advocacy, campaigns, development programs and to respond swiftly to emergencies. Each of the 20 Affiliates is an independent organization with its own areas of activity and work contributing its own strengths and expertise. Additionally, Oxfam has three Public Engagement Offices that are located in three countries (South Korea, Sweden and Argentina) that raise awareness and funds for Oxfam's work.

Source: Oxfam International. (2020). *Oxfam.* Retrieved from: https://www.oxfam.org/en

Search Strategy

1. The list of countries where Oxfam operates was obtained from the Oxfam International website under the "Working worldwide" page. Based on this list of countries, the researcher clicked on each country specific link. The researcher was often redirected to the country's profile within the Oxfam International website. In some cases, the profile further redirected the researcher to the country specific website or the country's profile within a regional website.

2. Once the country profile, Country Office or Regional Offices' website was identified, the researcher searched for information related to COVID-19 and food security. This information was found:
    a. In one centralized spot highlighting the Country Office's COVID-19 response;
    b. In the news, press release, or blog posts section of the website with stories highlighting specific interventions addressing COVI-19; and
    c. By using a targeted search of food security interventions in the context of COVID-19 (keywords searched: "COVID" OR "corona" AND "food" OR "seed" OR "nutrition" OR "agriculture" OR "meal" OR "ration")

3. To complement this search, all Oxfam Affiliate and Public Engagement Offices websites were also searched with interventions identified in:
    a. One centralized spot highlighting the Affiliate's COVID-19 response;
    b. In the news, press release, or blog posts section of the website; and
    c. By using a targeted search of food security interventions in the context of COVID-19 (keywords searched: "COVID" OR "corona" AND "food" OR "seed" OR "nutrition" OR "agriculture" OR "meal" OR "ration")

4. As a final complementary step, a targeted Google search was conducted to identify any remaining interventions not captured in the first two search methods. (keywords to search: "Oxfam" AND "COVID" OR "corona" AND "food" OR "seed" OR "nutrition" OR "agriculture" OR "meal" or "ration")

International Committee of the Red Cross

Purpose: The International Committee of the Red Cross's (ICRC) mission is to protect the lives and dignity of victims of armed conflict and other situations of violence and to provide them with assistance.

Structure of Organization: Established in 1863, the ICRC is at the origin of the Geneva Conventions and the International Red Cross and Red Crescent Movement. The Red Cross and Red Crescent Movement is made up of three parts: the International Committee of the Red Cross (ICRC) the International Federation of Red Cross and Red Crescent Societies (IFRC) and 191 National Red Cross and Red Crescent Societies around the world. The ICRC helps victims of armed conflict and internal crises and co-ordinates the work of National Societies in these situations.

Source: International Committee of the Red Cross. (2020). *ICRC.* Retrieved from: https://www.icrc.org/en

Search Strategy:

1. The list of countries where the ICRC operates was identified on the ICRC International website under the "Where We Work" page. Based on this list of countries, the researchers clicked on each LMIC specific link, which redirected them to the country's profile within the ICRC international website.

2. Once the country specific webpages within the international website were identified, the researcher searched for information related to COVID-19 and food security. This information was found:
    a. On the main page as a featured topic
    b. As news articles highlighting specific interventions addressing Food Security and COVID-19
    c. Within reports or articles released by the ICRC
    d. Each source was examined using a targeted search for food security interventions in the context of COVID-19 (keywords searched "COVID" OR "corona" AND "food" OR "seed" OR "nutrition" OR "agriculture" OR "meal" OR "ration"

3. An additional targeted search of COVID-19 specific interventions were conducted from the ICRC international website under the "Coronavirus: COVID-19 Pandemic" page. This information was found:
    a. In news articles or reports that were not captured in the country level screening, which typically included continent level summaries
    b. Each source was examined using a targeted search for security interventions in the context of COVID-19 (keywords searched: "COVID" OR "corona" AND "food" OR "seed" OR "nutrition" OR "agriculture" OR "meal" OR "ration"

4. As a final complementary step, a targeted Google search was conducted to identify any remaining interventions not captured in the first two search methods. (keywords searched: "ICRC" AND "COVID" OR "corona" AND "food" OR "seed" OR "nutrition" OR "agriculture" OR "meal" OR "ration")

International Federation of Red Cross and Red Crescent Societies

Purpose: The International Federation of Red Cross and Red Crescent Societies (IFRC) mission is to improve the lives of vulnerable people by mobilizing the power of humanity specifically following natural and man-made disasters in non-conflict situations.

Structure of Organization: The IFRC is a key part of the International Red Cross and Red Crescent Movement. The Red Cross and Red Crescent Movement is made up of three parts: the International Committee of the Red Cross (ICRC) the International Federation of Red Cross and Red Crescent Societies (IFRC) and 191 National Red Cross and Red Crescent Societies around the world. The International Federation of Red Cross and Red Crescent Societies (IFRC) co-ordinates international relief provided by National Societies for victims of natural disasters, and for refugees and displaced persons outside conflict zones. It also supports National Societies with their own development, helping them plan and implement disaster responses and development projects for vulnerable people in their local communities.

Source: The International Federation of Red Cross and Red Crescent Societies. (2020). *The International Red Cross and Red Crescent Movement.* Retrieved from: https://www.ifrc.org/en/who-we-are/the-movement/

Search Strategy

1. The list of countries where IFRC operates was identified on the IFRC international website under the "Where We Work" page by clicking the list of continents and then the individual national agencies

2. Once the country specific webpages within the international website were identified, researchers searched for information related to COVID-19 and food security. This information was found:
    a. As news articles highlighting specific interventions addressing Food Security and COVID-19
    b. In articles and reports released by the IFRC
    c. Each source was examined using a targeted search for food security interventions in the context of COVID-19 (keywords searched: "COVID" OR "corona" AND "food" OR "seed" OR "nutrition" OR "agriculture" OR "meal" OR "ration")

3. An additional targeted search of COVID-19 specific interventions were conducted from the IFRC International website under the "Global: Novel Coronavirus Disease (COVID-19) Outbreak" page. This information was found:
    a. In news articles or reports that were not captured in the country level screening
    b. In the most recent operations update, the "Three Month Update" released on May 28th, 2020
    c. Each source was examined using a targeted search for food security interventions in the context of COVID-19 (keywords searched: "COVID" OR "corona" AND "food" OR "seed" OR "nutrition" OR "agriculture" OR "meal" OR "ration")

4. As a final complementary step, a targeted Google search was conducted to identify any remaining interventions not captured in the first two search methods. (keywords searched: "IFRC" AND "COVID" OR "corona" AND "food" OR "seed" OR "nutrition" OR "agriculture" OR "meal" OR "ration"

World Vision

Purpose: World Vision is an international partnership of Christians whose mission is to work with the poor and oppressed to promote human transformation, seek justice and achieve faith-based objectives.

Structure of Organization: Established in 1977, World Vision International provides global coordination for the World Vision offices worldwide (referred to as the 'Partnerships'). They ensure that global standards and policies are pursued and serve as the implementing entity in several countries. World Vision is composed of a global center, support offices, and field offices. The Global Center is the office for World Vision International, and where global coordination functions are operationalized. The office is located in London, England. Support Offices fundraise to allow World Vision to implement programs worldwide, and Field Offices coordinate local agencies and community members to implement programs in the community setting.

Source: World Vision International. (2020). *World Vision.* Retrieved from: https://www.wvi.org/

Search Strategy

1. The list of countries where World Vision operates was obtained from the World Vision International website under the "All Locations" page. Each country (Field Office) where World Vision operated was searched for relevant interventions by following the country specific link from the World Vision International website. The link often redirected the researcher to either the country's profile within the World Vision International website or the Field Office website.

2. Once the country profile or Field Offices' website was identified, the researcher searched for relevant information related to COVID-19 and food security. This information was found:
    a. In a centralized spot summarizing all the COVID-19-related interventions of the organization;
    b. In the news, press release, or blog posts section of the website with stories highlighting specific interventions addressing COVI-19; and
    c. By using a targeted search of food security interventions in the context of COVID-19 (keywords searched: "COVID" OR "corona" AND "food" OR "seed" OR "nutrition" OR "agriculture" OR "meal")

3. To complement this search, all World Vision Support Offices websites were also searched with interventions identified in:
    a. In one centralized spot highlighting the Affiliate's COVID-19 response;
    b. In the news, press release, or blog posts section of the website; and
    c. By using a targeted search of food security interventions in the context of COVID-19 (keywords searched: "COVID" OR "corona" AND "food" OR "seed" OR "nutrition" OR "agriculture" OR "meal")

4. As a final complementary step, a targeted Google search was conducted to identify any remaining interventions not captured in the first two search methods. (keywords to search: "World Vision" AND "COVID" OR "corona" AND "food" OR "seed" OR "nutrition" OR "agriculture" OR "meal")

United Nations Children's Fund

Purpose: UNICEF promotes the rights and wellbeing of children, and works to overcome the obstacles that poverty, violence, disease and discrimination place in a child's path. To implement their mission, UNICEF works in collaboration with partners in 190 countries. UNICEF focuses specifically on reaching vulnerable children including those living in fragile contexts, those living with a disability, those who are affected by rapid urbanization and those affected by environmental degradation. The agency takes a life-cycle based approach, recognizing the particular importance of early childhood development and adolescence.

Source: The United Nations Children's Fund. (2020). *UNICEF.* Retrieved from: https://www.unicef.org/

Search Strategy:

1. To identify related UNICEF interventions the following sources on UNICEF's official website were searched
    a. COVID-19 Response Page: Search for specific interventions related to food security or related links. The following sections of the COVID-19 Response Page was searched: latest, updates and features, tips and guidance for families, how UNICEF is helping, stories and features, media resources and related sources
    b. Situation Reports and Publications: Search situation reports and publications released between April 1st - May 31st for specific interventions related to food security in the context of COVID-19
    c. News or Blog Stories: Search news or blog stories between April 1st - May 31st for specific interventions related to food security in the context of COVID-19
    d. Country profiles: Search each country profile for specific interventions related to food security in the context of COVID-19.

2. A targeted search of UNICEF's official website was also done using the website's search function for food security interventions in the context of COVID-19 (keywords to search: "COVID" OR "corona" AND "food" OR "seed" OR "nutrition" OR "agriculture" OR "meal")

World Food Programme

Purpose: The WFP delivers food assistance in emergencies and works with communities to improve nutrition and build resilience. WFP focuses on emergency assistance, relief and rehabilitation, development aid and special operations, with an emphasis on providing food in conflict affected areas. WFP has more than 17,000 staff worldwide of whom over 90 percent are based in the countries where the agency provides assistance. WFP partners with more than 1,000 national and international NGOs to provide food assistance and tackle the underlying causes of hunger.

Source: The United Nations World Food Programme. (2020). *WFP.* Retrieved from: https://www.wfp.org/

Search Strategy:

1. To identify related WFP interventions the following sources on WFP's official website were searched
    e. COVID-19 Response Page: Search for specific interventions related to food security or related links
    f. Situation Reports and Publications: Search situation reports and publications released between April 1st - May 31st for specific interventions related to food security in the context of COVID-19
    g. News or Blog Stories: Search news or blog stories between April 1st - May 31st for specific interventions related to food security in the context of COVID-19
    h. Country profiles: Search each country profile for specific interventions related to food security in the context of COVID-19.

2. A targeted search of WFP's official website was also done using the website's search function for food security interventions in the context of COVID-19 (keywords to search: "covid" OR "corona" AND "food" OR "seed" OR "nutrition" OR "agriculture" OR "meal")

United Nations Refugee Agency

Purpose: UNHCR focuses on ensuring that everyone has the right to seek asylum and find safe refuge in another State, with the option to eventually return home, integrate or resettle. During times of displacement, UNHCR provides critical emergency assistance in the form of clean water, sanitation and healthcare, as well as shelter, blankets, household goods and food. The organization also arranges transport and assistance packages for people who return home, and income-generating projects for those who resettle.

Source: The United Nations Refugee Agency. (2020). *UNHCR.* Retrieved from: https://www.unhcr.org/

Search Strategy:

1. To identify related UNHCR interventions the following sources on UNHCR's official website were searched.
   a. The coronavirus outbreak page within the emergencies section of the UNHCR international website
   b. Situation reports and articles related to COVID-19 in the context of food security
   c. News stories highlighting specific COVID-19 interventions related to food security
   d. The most recent operations update, the three-month update, from May 28th 2020
   d. Each source was examined using a targeted search for food security interventions in the context of COVID-19 (keywords searched: "COVID" OR "corona" AND "food" OR "seed" OR "nutrition" OR "agriculture" OR "meal" OR "ration")

As a final complementary step, a targeted Google search was conducted to identify any remaining interventions not captured in the first two search methods. (keywords searched: "UNHCR" AND "COVID" OR "corona" AND "food" OR "seed" OR "nutrition" OR "agriculture" OR "meal" OR "ration"

Table S2. List of details extracted on food security interventions in the context of the COVID-19 pandemic

CATEGORY	DEFINITIONS AND NOTES
**List name of organization and/or country level office**	provide name of organization implementing intervention
**Continent**<br>1 = Asia<br>2 = Africa<br>3 = Europe<br>4 = North America<br>5 = South America<br>6 = Oceania	countries defined as being in more than on continent were categorized based on the continent in which the majority of the country is located
**List country**	only low-and-middle income countries as defined by the World Bank[a]
**List specific region, city, village**	provide name of region, city, village, or other area where intervention took place
**List scale**<br>1 = community	single district, city, town, village, refugee camp

2 = regional	any two places, province, or region within a country
3 = national	implementation at a national level (e.g. 'across country') is specifically stated
4 = undefined	no specific scale mentioned
**Provide brief description**	provide a short paragraph with the details of the intervention
**Program type**	
undefined	mutually exclusive, when program type is unspecified
distributing food aid	e.g. food assistance, in-kind food, food stuffs, food rations
implementing feeding programs	e.g. hot meals, soup kitchen, school feeding program
supporting local food production	e.g. agricultural inputs, seeds, resources for farmers
supporting food supply chain	specific mention of supply chain or intervention to increase the supply of food
providing cash transfer	e.g. cash assistance, cash provided, monetary rations
providing food voucher	e.g. voucher tied specifically to food/groceries
supporting food safety	specific mention of food safety; e.g. enhancing food safety through proper storage
nutrition program	explicit mention of nutrition program or intention of addressing nutrition through food; e.g. malnutrition intervention
monitoring, technical, and policy support	e.g. technical assistance, logistic support, operational guidance, policy recommendation
prepositioning	prepositioning and procuring of food for long term supply or other purposes
livelihood and income generation	e.g. livelihood opportunities, skill building, income generation, selling produce, community savings program, social protection program, and asset building
other	
define other	
**New/adapted/ongoing**	
1 = new program	emerged specifically in response to COVID
2 = adapted existing program	adapted program because of COVID
3 = ongoing	existing work still occurring in context of COVID, no changes
4 = undefined	mutually exclusive
**List intended beneficiaries**	
undefined	mutually exclusive, when intended beneficiaries of intervention is unspecified
undefined vulnerable population	mutually exclusive, when "vulnerable population" is the only descriptor given for the intended beneficiary
infants/children/youth and adolescents	e.g. children, malnourished children, infants, school aged children, adolescents, youth, etc.
women	e.g. pregnant women, mothers, female household heads, etc.
household/families	e.g. parents, vulnerable families and households, etc.
older adults/elderly	e.g. elderly people, older adults, seniors, elders, etc.
farmers	e.g. local producers, farmers, agricultural workers, etc.
migrants	e.g. migrant workers, groups in transit, returnees, etc.

refugees/internally displaced people	e.g. refugees from different areas, displacement, conflict, etc.
Indigenous Peoples	broadly defined Indigenous Peoples or mention of specific Indigenous groups; e.g. Mayan communities
low income/poor populations	e.g. poorest people, low income earners, etc.
people experiencing homelessness/living in the streets	e.g. homelessness, living in the streets, etc.
frontline workers - HCP	e.g. physicians, nurses, healthcare workers, etc.
frontline workers - volunteers	e.g. frontline civil society, volunteers, etc.
people with underlying medical conditions	e.g. patients TB, HIV, cancer, chronic disease, etc.
people living with disabilities	broadly defined people living with a disability or specific mention of disability; e.g. orphanage for children with visual impairments
rural/remote areas	people living in rural or remote areas
informal sector workers/daily wage earners	e.g. workers, informal sector, daily wage earners, day labourers, etc.
other	
**Aspect of COVID-19 addressed by intervention**	
undefined	mutually exclusive
unemployment/reduced cash flow	job loss or less work directly related to COVID-19
school and feeding program closures	school/feeding program closure as a result of COVID-19
government measures restricting mobility	government enforced measures designed to restrict mobility in response to COVID; e.g. quarantine facility, lockdown, quarantine, etc.
disrupted supply chain	mention of supply chain disruption; e.g. trade restrictions, closed markets, food delayed in transit, etc.
increased cost of goods	mention of increased cost of good as a result of COVID-19
environmental vulnerabilities	mention of natural disaster (e.g. flood, cyclone), climate change, disruption of farming activities (e.g. drought, locust invasion, lean season)
displacement/conflict	mention of displacement or conflict (political, armed or others)
existing food insecurity/malnutrition	specific mention of food insecurity or malnutrition problems
other	use to indicate if a specific vulnerability is exacerbated (e.g. existing food insecurity, malnutrition problems, weak health systems, limited access to resources, conflict or displacement) or if another factor is mentioned
**Pillars of food security**[b, c]	
availability	enough nutritious food of sufficient quality needs to be available to people for their consumption
· production	how much and what types of food are available through food that is produced and stored locally - e.g. providing seeds to farmers
· distribution	how is food made available (physically moved), in what form, when, and to whom - e.g. food aid

· exchange	how much of food that is available can be obtained through exchange mechanisms such as barter, trade, purchase, or loans - e.g. support small scale producers sell their products
access	individuals and households must be able to acquire sufficient food to be able to eat a healthy, nutritious diet, or have access to sufficient resources needed to grow their own food (e.g. land).
· affordability	the ability of individuals, households or communities to afford the price of food or land for producing food, relative to their incomes - e.g. cash transfer
· allocation	the economic, social and political mechanisms governing when, where, and how food can be accessed by consumers and on what terms. For example, food may be unequally allocated according to age and gender within households
· preference	social, religious, and cultural norms and values that influence consumer demand for certain types of food (e.g. religious prohibitions or the desire to follow a specific dietary pattern such as vegetarianism)
utilization	people must have access to a sufficient quantity and diversity of foods to meet their nutritional needs but must also be able to eat and properly metabolise such food.
· nutritional value	the nutritional value provided by the foods that are consumed, as measured in calories, vitamins, protein, and various micronutrients (e.g. iron, iodine, vitamin A) - e.g. malnutrition and other nutrition interventions
· health status	the effect of disease (e.g. HIV/AIDS or diarrhoea) on the ability to consume the food and absorb and metabolise its nutrients - e.g. intervention specifically related to disease
· food safety	access to food free from food spoilage or from toxic contamination introduction during the producing, processing, packaging, distribution or marketing of food; and from food-borne diseases such as salmonella.
· preparation and consumption	the resources (e.g. cooking tools and fuel), knowledge and ability to prepare and consume food in a healthy and hygienic way.
· cultural acceptability	the stability of food based on environmental factors such as extreme weather events and climate change - e.g. cash transfer with mention of allowing people to choose what food suited them
stability	food may be available and accessible to people who are able to utilise it effectively, but to avoid increases in malnutrition and in order for people not to feel insecure, this state of affairs needs to be enduring rather than temporary or subject to fluctuations.
· environmental stability	the stability of food based on environmental factors such as extreme weather events and climate change - e.g. if intervention is in response to COVID and an environmental concern like floods or locust
· stable supply	the fluctuation in food supply over time - e.g. if stable supply is specifically mentioned
**Partners**	
undefined	mutually exclusive
other NGO	any other non-governmental organization
local government	local, district, municipal, mayor, chief, council (does not include community leader broadly defined)
regional government	regional, provincial

national government	any mention of state, ministry, government, etc. that is not specifically described as local, municipal, regional, etc.
faith-based organization	any mention of church, FBO, perish, mosque, temple, or other religious group
health workers	CHWs, nurses, doctors, health clinics, etc.
community volunteers	civil society
UN agency	any UN agency
business	any partner described as a business or other corporation
other	if another partner is mentioned without fitting into the above category, even if it is broad like 'other partners' code and define as 'other'
**Implementation**	describe any aspects of implementation - e.g. double rations, increased delivery points, including somethings described as food safety (e.g. hand hygiene to avoid spreading COVID)
**Implementation challenges**	describe any challenges with implementation
**Source**	provide link to website
**Date sourced**	provide date intervention was identified

[a] World Bank. (2020). *Data.* Retrieved from: https://datahelpdesk.worldbank.org/knowledgebase/articles/906519-world-bank-country-and-lending-groups
[b] FCRN. (2020). *What is Food Security.* Retrieve from: https://www.foodsource.org.uk/building-blocks/what-food-security
[c] FAO. (2008). *An Introduction to the Basic Concepts of Food Security*. Retrieved from: http://www.fao.org/3/a-al936e.pdf

Table S3. Characteristics of food security interventions implemented in response to the COVID-19 pandemic by INGOs and UN agencies in low-and-middle income countries from Dec 31, 2019 to May 31, 2020 (*n*=287)

	WORLD VISION		OXFAM		ICRC		IFRC		CARE		UNHCR		UNICEF		WFP		INGO		UN AGENCIES		OVERALL	
	n	%	n	%	n	%	n	%	n	%	n	%	n	%	n	%	n	%	n	%	n	%
	50	17.42	13	4.53	11	3.83	46	16.03	18	6.27	43	14.98	36	12.54	70	24.39	138	48.08	149	51.92	287	100.0 0
CONTINENT																						
Asia	20	40.00	9	69.23	4	36.36	12	26.09	7	38.89	7	16.28	16	44.44	34	48.57	52	37.68	57	38.26	109	37.98
Africa	16	32.00	2	15.38	1	9.09	4	8.70	3	16.67	22	51.16	11	30.56	36	51.43	26	18.84	69	46.31	95	33.10
Europe	1	2.00	0	0.00	2	18.18	17	36.96	0	0.00	3	6.98	2	5.56	0	0.00	20	14.49	5	3.36	25	8.71
North America	4	8.00	1	7.69	0	0.00	6	13.04	7	38.89	0	0.00	2	5.56	0	0.00	18	13.04	2	1.34	20	6.97
South America	9	18.00	1	7.69	4	36.36	7	15.22	1	5.56	11	25.58	5	13.89	0	0.00	22	15.94	16	10.74	38	13.24
Oceania	0	0.00	0	0.00	0	0.00	0	0.00	0	0.00	0	0.00	0	0.00	0	0.00	0	0.00	0	0.00	0	0.00
COUNTRY																						
Afghanistan	1	2.00	0	0.00	0	0.00	0	0.00	0	0.00	0	0.00	1	2.78	1	1.43	1	0.72	2	1.34	3	1.05
Albania	1	2.00	0	0.00	0	0.00	1	2.17	0	0.00	0	0.00	0	0.00	0	0.00	2	1.45	0	0.00	2	0.70
Algeria	0	0.00	0	0.00	0	0.00	1	2.17	0	0.00	0	0.00	0	0.00	3	4.29	1	0.72	3	2.01	4	1.39
Angola	0	0.00	0	0.00	0	0.00	0	0.00	0	0.00	0	0.00	1	2.78	0	0.00	0	0.00	1	0.67	1	0.35
Argentina	0	0.00	0	0.00	0	0.00	1	2.17	0	0.00	0	0.00	2	5.56	0	0.00	1	0.72	2	1.34	3	1.05
Armenia	1	2.00	0	0.00	0	0.00	1	2.17	0	0.00	0	0.00	0	0.00	1	1.43	2	1.45	1	0.67	3	1.05
Azerbaijan	0	0.00	0	0.00	0	0.00	1	2.17	0	0.00	0	0.00	0	0.00	0	0.00	1	0.72	0	0.00	1	0.35
Bangladesh	2	4.00	2	15.38	0	0.00	1	2.17	0	0.00	0	0.00	1	2.78	3	4.29	5	3.62	4	2.68	9	3.14
Belarus	0	0.00	0	0.00	0	0.00	2	4.35	0	0.00	0	0.00	0	0.00	0	0.00	2	1.45	0	0.00	2	0.70
Belize	0	0.00	0	0.00	0	0.00	1	2.17	0	0.00	0	0.00	1	2.78	0	0.00	1	0.72	1	0.67	2	0.70
Bhutan	0	0.00	0	0.00	0	0.00	0	0.00	0	0.00	0	0.00	5	13.89	1	1.43	0	0.00	6	4.03	6	2.09
Bolivia	1	2.00	0	0.00	0	0.00	1	2.17	0	0.00	0	0.00	1	2.78	0	0.00	2	1.45	1	0.67	3	1.05
Bosnia and Herzegovina	1	2.00	0	0.00	0	0.00	1	2.17	0	0.00	0	0.00	0	0.00	0	0.00	2	1.45	0	0.00	2	0.70
Botswana	0	0.00	0	0.00	0	0.00	0	0.00	0	0.00	0	0.00	0	0.00	0	0.00	0	0.00	0	0.00	0	0.00
Brazil	4	8.00	1	7.69	0	0.00	0	0.00	0	0.00	3	6.98	0	0.00	0	0.00	5	3.62	3	2.01	8	2.79
Bulgaria	0	0.00	0	0.00	0	0.00	0	0.00	0	0.00	0	0.00	0	0.00	0	0.00	0	0.00	0	0.00	0	0.00
Burkina Faso	0	0.00	0	0.00	0	0.00	0	0.00	0	0.00	0	0.00	0	0.00	0	0.00	0	0.00	0	0.00	0	0.00
Burundi	0	0.00	0	0.00	0	0.00	0	0.00	0	0.00	0	0.00	0	0.00	0	0.00	0	0.00	0	0.00	0	0.00
Cambodia	0	0.00	0	0.00	0	0.00	1	2.17	0	0.00	0	0.00	0	0.00	0	0.00	1	0.72	0	0.00	1	0.35

Central African Republic	0	0.00	1	7.69	0	0.00	0	0.00	0	0.00	0	0.00	0	0.00	0	0.00	1	0.72	0	0.00	1	0.35
Chad	1	2.00	0	0.00	0	0.00	0	0.00	0	0.00	0	0.00	0	0.00	4	5.71	1	0.72	4	2.68	5	1.74
China	0	0.00	0	0.00	0	0.00	0	0.00	0	0.00	0	0.00	0	0.00	1	1.43	0	0.00	1	0.67	1	0.35
Colombia	2	4.00	0	0.00	1	9.09	0	0.00	0	0.00	1	2.33	0	0.00	0	0.00	3	2.17	1	0.67	4	1.39
Costa Rica	1	2.00	0	0.00	0	0.00	1	2.17	1	5.56	0	0.00	0	0.00	0	0.00	3	2.17	0	0.00	3	1.05
Cote d'Ivoire	0	0.00	0	0.00	0	0.00	0	0.00	0	0.00	0	0.00	0	0.00	2	2.86	0	0.00	2	1.34	2	0.70
Democratic Republic of Congo	0	0.00	0	0.00	1	9.09	0	0.00	0	0.00	2	4.65	0	0.00	0	0.00	1	0.72	2	1.34	3	1.05
Djibouti	0	0.00	0	0.00	0	0.00	0	0.00	0	0.00	0	0.00	0	0.00	2	2.86	0	0.00	2	1.34	2	0.70
Dominica	0	0.00	0	0.00	0	0.00	1	2.17	0	0.00	0	0.00	0	0.00	0	0.00	1	0.72	0	0.00	1	0.35
Dominican Republic	1	2.00	0	0.00	0	0.00	1	2.17	0	0.00	0	0.00	0	0.00	0	0.00	2	1.45	0	0.00	2	0.70
Ecuador	0	0.00	0	0.00	0	0.00	1	2.17	0	0.00	3	6.98	0	0.00	0	0.00	1	0.72	3	2.01	4	1.39
Egypt	0	0.00	0	0.00	0	0.00	1	2.17	0	0.00	0	0.00	0	0.00	3	4.29	1	0.72	3	2.01	4	1.39
El Salvador	0	0.00	0	0.00	0	0.00	1	2.17	0	0.00	0	0.00	0	0.00	0	0.00	1	0.72	0	0.00	1	0.35
Equatorial Guinea	0	0.00	0	0.00	0	0.00	0	0.00	0	0.00	0	0.00	1	2.78	0	0.00	0	0.00	1	0.67	1	0.35
Eswatini	0	0.00	0	0.00	0	0.00	0	0.00	0	0.00	0	0.00	0	0.00	0	0.00	0	0.00	0	0.00	0	0.00
Ethiopia	0	0.00	0	0.00	0	0.00	0	0.00	0	0.00	0	0.00	1	2.78	0	0.00	0	0.00	1	0.67	1	0.35
Georgia	0	0.00	0	0.00	0	0.00	1	2.17	0	0.00	0	0.00	1	2.78	0	0.00	1	0.72	1	0.67	2	0.70
Ghana	0	0.00	0	0.00	0	0.00	0	0.00	0	0.00	0	0.00	0	0.00	0	0.00	0	0.00	0	0.00	0	0.00
Guatemala	0	0.00	1	7.69	0	0.00	0	0.00	1	5.56	0	0.00	0	0.00	0	0.00	2	1.45	0	0.00	2	0.70
Guinea	0	0.00	0	0.00	0	0.00	0	0.00	0	0.00	0	0.00	0	0.00	1	1.43	0	0.00	1	0.67	1	0.35
Guinea-Bissau	0	0.00	0	0.00	0	0.00	0	0.00	0	0.00	1	2.33	0	0.00	0	0.00	0	0.00	1	0.67	1	0.35
Guyana	0	0.00	0	0.00	0	0.00	1	2.17	0	0.00	0	0.00	1	2.78	0	0.00	1	0.72	1	0.67	2	0.70
Haiti	1	2.00	0	0.00	0	0.00	0	0.00	2	11.11	0	0.00	0	0.00	0	0.00	3	2.17	0	0.00	3	1.05
Honduras	0	0.00	0	0.00	0	0.00	1	2.17	2	11.11	0	0.00	1	2.78	0	0.00	3	2.17	1	0.67	4	1.39
India	2	4.00	4	30.77	0	0.00	0	0.00	3	16.67	0	0.00	2	5.56	4	5.71	9	6.52	6	4.03	15	5.23
Indonesia	2	4.00	0	0.00	0	0.00	0	0.00	0	0.00	1	2.33	1	2.78	1	1.43	2	1.45	3	2.01	5	1.74
Iran	0	0.00	0	0.00	0	0.00	1	2.17	0	0.00	0	0.00	0	0.00	2	2.86	1	0.72	2	1.34	3	1.05
Iraq	0	0.00	1	7.69	1	9.09	1	2.17	0	0.00	0	0.00	0	0.00	3	4.29	3	2.17	3	2.01	6	2.09
Jamaica	0	0.00	0	0.00	0	0.00	1	2.17	0	0.00	0	0.00	0	0.00	0	0.00	1	0.72	0	0.00	1	0.35
Jordan	0	0.00	0	0.00	0	0.00	1	2.17	0	0.00	2	4.65	1	2.78	2	2.86	1	0.72	5	3.36	6	2.09
Kazakhstan	0	0.00	0	0.00	0	0.00	1	2.17	0	0.00	0	0.00	0	0.00	0	0.00	1	0.72	0	0.00	1	0.35
Kenya	3	6.00	0	0.00	0	0.00	0	0.00	1	5.56	1	2.33	0	0.00	1	1.43	4	2.90	2	1.34	6	2.09
Kyrgyzstan	0	0.00	0	0.00	0	0.00	1	2.17	0	0.00	0	0.00	0	0.00	2	2.86	1	0.72	2	1.34	3	1.05
Laos	0	0.00	0	0.00	0	0.00	0	0.00	0	0.00	0	0.00	0	0.00	0	0.00	0	0.00	0	0.00	0	0.00
Lebanon	1	2.00	0	0.00	0	0.00	0	0.00	1	5.56	1	2.33	0	0.00	1	1.43	2	1.45	2	1.34	4	1.39
Lesotho	0	0.00	0	0.00	0	0.00	0	0.00	0	0.00	0	0.00	0	0.00	0	0.00	0	0.00	0	0.00	0	0.00

Liberia	0	0.00	0	0.00	0	0.00	1	2.17	0	0.00	1	2.33	0	0.00	1	1.43	1	0.72	2	1.34	3	1.05
Libya	0	0.00	0	0.00	0	0.00	0	0.00	0	0.00	2	4.65	0	0.00	1	1.43	0	0.00	3	2.01	3	1.05
Madagascar	0	0.00	0	0.00	0	0.00	0	0.00	1	5.56	0	0.00	1	2.78	0	0.00	1	0.72	1	0.67	2	0.70
Malawi	0	0.00	0	0.00	0	0.00	0	0.00	0	0.00	0	0.00	1	2.78	0	0.00	0	0.00	1	0.67	1	0.35
Malaysia	1	2.00	0	0.00	0	0.00	0	0.00	0	0.00	0	0.00	0	0.00	0	0.00	1	0.72	0	0.00	1	0.35
Mali	0	0.00	0	0.00	0	0.00	0	0.00	0	0.00	0	0.00	0	0.00	2	2.86	0	0.00	2	1.34	2	0.70
Mauritania	0	0.00	0	0.00	0	0.00	0	0.00	0	0.00	2	4.65	0	0.00	0	0.00	0	0.00	2	1.34	2	0.70
Mauritius	0	0.00	0	0.00	0	0.00	0	0.00	0	0.00	0	0.00	0	0.00	0	0.00	0	0.00	0	0.00	0	0.00
Mexico	1	2.00	0	0.00	0	0.00	0	0.00	0	0.00	0	0.00	0	0.00	0	0.00	1	0.72	0	0.00	1	0.35
Mongolia	2	4.00	0	0.00	0	0.00	0	0.00	0	0.00	0	0.00	0	0.00	0	0.00	2	1.45	0	0.00	2	0.70
Montenegro	0	0.00	0	0.00	0	0.00	1	2.17	0	0.00	0	0.00	0	0.00	0	0.00	1	0.72	0	0.00	1	0.35
Morocco	0	0.00	0	0.00	0	0.00	0	0.00	0	0.00	0	0.00	0	0.00	0	0.00	0	0.00	0	0.00	0	0.00
Mozambique	1	2.00	0	0.00	0	0.00	0	0.00	0	0.00	0	0.00	0	0.00	0	0.00	1	0.72	0	0.00	1	0.35
Myanmar	1	2.00	0	0.00	1	9.09	1	2.17	0	0.00	0	0.00	1	2.78	1	1.43	3	2.17	2	1.34	5	1.74
Namibia	0	0.00	0	0.00	0	0.00	0	0.00	0	0.00	0	0.00	0	0.00	0	0.00	0	0.00	0	0.00	0	0.00
Nepal	1	2.00	0	0.00	0	0.00	0	0.00	0	0.00	0	0.00	1	2.78	1	1.43	1	0.72	2	1.34	3	1.05
Nicaragua	0	0.00	0	0.00	0	0.00	0	0.00	1	5.56	0	0.00	0	0.00	0	0.00	1	0.72	0	0.00	1	0.35
Niger	0	0.00	0	0.00	0	0.00	0	0.00	0	0.00	1	2.33	1	2.78	2	2.86	0	0.00	4	2.68	4	1.39
Nigeria	0	0.00	0	0.00	0	0.00	1	2.17	0	0.00	2	4.65	1	2.78	0	0.00	1	0.72	3	2.01	4	1.39
North Macedonia	0	0.00	0	0.00	0	0.00	1	2.17	0	0.00	0	0.00	0	0.00	0	0.00	1	0.72	0	0.00	1	0.35
Pakistan	0	0.00	0	0.00	0	0.00	1	2.17	0	0.00	1	2.33	0	0.00	1	1.43	1	0.72	2	1.34	3	1.05
Palestine	0	0.00	0	0.00	0	0.00	0	0.00	0	0.00	0	0.00	0	0.00	3	4.29	0	0.00	3	2.01	3	1.05
Paraguay	0	0.00	0	0.00	1	9.09	1	2.17	0	0.00	0	0.00	0	0.00	0	0.00	2	1.45	0	0.00	2	0.70
Peru	1	2.00	0	0.00	1	9.09	1	2.17	1	5.56	3	6.98	0	0.00	0	0.00	4	2.90	3	2.01	7	2.44
Philippines	5	10.00	2	15.38	1	9.09	2	4.35	2	11.11	0	0.00	0	0.00	0	0.00	12	8.70	0	0.00	12	4.18
Republic of Congo	0	0.00	0	0.00	0	0.00	0	0.00	0	0.00	0	0.00	0	0.00	1	1.43	0	0.00	1	0.67	1	0.35
Romania	0	0.00	0	0.00	0	0.00	2	4.35	0	0.00	0	0.00	1	2.78	0	0.00	2	1.45	1	0.67	3	1.05
Russia	0	0.00	0	0.00	0	0.00	2	4.35	0	0.00	0	0.00	0	0.00	0	0.00	2	1.45	0	0.00	2	0.70
Rwanda	0	0.00	0	0.00	0	0.00	0	0.00	0	0.00	0	0.00	0	0.00	1	1.43	0	0.00	1	0.67	1	0.35
Saint Lucia	0	0.00	0	0.00	0	0.00	0	0.00	0	0.00	0	0.00	0	0.00	0	0.00	0	0.00	0	0.00	0	0.00
Sao Tome and Principe	0	0.00	0	0.00	0	0.00	0	0.00	0	0.00	0	0.00	0	0.00	0	0.00	0	0.00	0	0.00	0	0.00
Senegal	1	2.00	0	0.00	0	0.00	0	0.00	0	0.00	0	0.00	0	0.00	2	2.86	1	0.72	2	1.34	3	1.05
Serbia	0	0.00	0	0.00	0	0.00	1	2.17	0	0.00	1	2.33	0	0.00	0	0.00	1	0.72	1	0.67	2	0.70
Sierra Leone	0	0.00	0	0.00	0	0.00	0	0.00	0	0.00	0	0.00	0	0.00	1	1.43	0	0.00	1	0.67	1	0.35
Somalia	0	0.00	0	0.00	0	0.00	0	0.00	0	0.00	1	2.33	2	5.56	0	0.00	0	0.00	3	2.01	3	1.05
South Africa	0	0.00	0	0.00	0	0.00	0	0.00	0	0.00	1	2.33	0	0.00	0	0.00	0	0.00	1	0.67	1	0.35

South Sudan	4	8.00	0	0.00	0	0.00	0	0.00	1	5.56	3	6.98	1	2.78	1	1.43	5	3.62	5	3.36	10	3.48
Sri Lanka	1	2.00	0	0.00	0	0.00	0	0.00	0	0.00	0	0.00	1	2.78	2	2.86	1	0.72	3	2.01	4	1.39
Sudan	2	4.00	0	0.00	0	0.00	0	0.00	0	0.00	0	0.00	0	0.00	0	0.00	2	1.45	0	0.00	2	0.70
Syria	0	0.00	0	0.00	0	0.00	1	2.17	0	0.00	1	2.33	0	0.00	1	1.43	1	0.72	2	1.34	3	1.05
Tajikistan	0	0.00	0	0.00	0	0.00	1	2.17	0	0.00	0	0.00	0	0.00	0	0.00	1	0.72	0	0.00	1	0.35
Tanzania	1	2.00	0	0.00	0	0.00	0	0.00	0	0.00	0	0.00	0	0.00	1	1.43	1	0.72	1	0.67	2	0.70
Timor Leste	0	0.00	0	0.00	0	0.00	0	0.00	0	0.00	0	0.00	1	2.78	1	1.43	0	0.00	2	1.34	2	0.70
Tunisia	0	0.00	0	0.00	0	0.00	0	0.00	0	0.00	2	4.65	0	0.00	2	2.86	0	0.00	4	2.68	4	1.39
Turkey	0	0.00	0	0.00	0	0.00	1	2.17	0	0.00	1	2.33	0	0.00	1	1.43	1	0.72	2	1.34	3	1.05
Uganda	2	4.00	0	0.00	0	0.00	0	0.00	0	0.00	2	4.65	0	0.00	1	1.43	2	1.45	3	2.01	5	1.74
Ukraine	0	0.00	0	0.00	2	18.18	1	2.17	0	0.00	2	4.65	1	2.78	0	0.00	3	2.17	3	2.01	6	2.09
Venezuela	1	2.00	0	0.00	1	9.09	0	0.00	0	0.00	1	2.33	1	2.78	0	0.00	2	1.45	2	1.34	4	1.39
Vietnam	0	0.00	0	0.00	0	0.00	0	0.00	0	0.00	0	0.00	0	0.00	0	0.00	0	0.00	0	0.00	0	0.00
Yemen	0	0.00	0	0.00	1	9.09	0	0.00	1	5.56	0	0.00	0	0.00	1	1.43	2	1.45	1	0.67	3	1.05
Zambia	0	0.00	0	0.00	0	0.00	0	0.00	0	0.00	0	0.00	0	0.00	3	4.29	0	0.00	3	2.01	3	1.05
Zimbabwe	0	0.00	1	7.69	0	0.00	0	0.00	0	0.00	1	2.33	1	2.78	1	1.43	1	0.72	3	2.01	4	1.39
SCALE																					
community	17	34.00	5	38.46	1	9.09	7	15.22	2	11.11	12	27.91	4	11.11	7	10.00	32	23.19	23	15.44	55	19.16
regional	19	38.00	5	38.46	5	45.45	7	15.22	9	50.00	9	20.93	8	22.22	25	35.71	45	32.61	42	28.19	87	30.31
national	4	8.00	0	0.00	0	0.00	14	30.43	0	0.00	3	6.98	12	33.33	6	8.57	18	13.04	21	14.09	39	13.59
undefined	10	20.00	3	23.08	5	45.45	18	39.13	7	38.89	19	44.19	12	33.33	32	45.71	43	31.16	63	42.28	106	36.93
PROGRAM TYPE[a]																					
distributing food aid	31	62.00	9	69.23	10	90.91	40	86.96	13	72.22	20	46.51	7	19.44	41	58.57	103	74.64	68	45.64	171	59.58
providing cash transfer	8	16.00	2	15.38	1	9.09	4	8.70	5	27.78	21	48.84	3	8.33	18	25.71	20	14.49	42	28.19	62	21.60
nutrition program	8	16.00	0	0.00	0	0.00	0	0.00	1	5.56	1	2.33	18	50.00	8	11.43	9	6.52	27	18.12	36	12.54
implementing feeding programs	3	6.00	1	7.69	1	9.09	8	17.39	1	5.56	3	6.98	2	5.56	8	11.43	14	10.14	13	8.72	27	9.41
monitoring, technical, and policy support	1	2.00	2	15.38	0	0.00	0	0.00	0	0.00	2	4.65	10	27.78	9	12.86	3	2.17	21	14.09	24	8.36
supporting local food production	9	18.00	1	7.69	0	0.00	1	2.17	1	5.56	3	6.98	3	8.33	3	4.29	12	8.70	9	6.04	21	7.32
supporting food supply chain	1	2.00	2	15.38	0	0.00	0	0.00	1	5.56	0	0.00	3	8.33	6	8.57	4	2.90	9	6.04	13	4.53
providing food voucher	4	8.00	0	0.00	0	0.00	1	2.17	2	11.11	0	0.00	0	0.00	3	4.29	7	5.07	3	2.01	10	3.48
livelihood and income generation	4	8.00	1	7.69	0	0.00	0	0.00	0	0.00	0	0.00	1	2.78	4	5.71	5	3.62	5	3.36	10	3.48
prepositioning and procuring	0	0.00	0	0.00	0	0.00	0	0.00	0	0.00	0	0.00	0	0.00	5	7.14	0	0.00	5	3.36	5	1.74
food safety	0	0.00	0	0.00	0	0.00	0	0.00	0	0.00	0	0.00	1	2.78	2	2.86	0	0.00	3	2.01	3	1.05

other	4	8.00	1	7.69	1	9.09	6	13.04	0	0.00	2	4.65	1	2.78	0	0.00	12	8.70	3	2.01	15	5.23
undefined	0	0.00	0	0.00	0	0.00	0	0.00	1	5.56	0	0.00	0	0.00	0	0.00	1	0.72	0	0.00	1	0.35
NEW/ADAPTED/ONGOING																				
new program	25	50.00	6	46.15	6	54.55	27	58.70	7	38.89	24	55.81	13	36.11	17	24.29	71	51.45	54	36.24	125	43.55
adapted existing program	18	36.00	4	30.77	1	9.09	4	8.70	7	38.89	10	23.26	17	47.22	31	44.29	34	24.64	58	38.93	92	32.06
ongoing	3	6.00	0	0.00	2	18.18	6	13.04	1	5.56	5	11.63	5	13.89	18	25.71	12	8.70	28	18.79	40	13.94
undefined	4	8.00	3	23.08	2	18.18	9	19.57	3	16.67	4	9.30	1	2.78	4	5.71	21	15.22	9	6.04	30	10.45
INTENDED BENEFICIARIES[a]																				
families	18	36.00	5	38.46	0	0.00	14	30.43	9	50.00	10	23.26	8	22.22	16	22.86	46	33.33	34	22.82	80	27.87
infants/children/youth	15	30.00	0	0.00	2	18.18	3	6.52	3	16.67	1	2.33	19	52.78	23	32.86	23	16.67	43	28.86	66	23.00
refugees/internally displaced people	9	18.00	2	15.38	3	27.27	2	4.35	3	16.67	30	69.77	1	2.78	13	18.57	19	13.77	44	29.53	63	21.95
migrants	7	14.00	3	23.08	2	18.18	6	13.04	2	11.11	3	6.98	1	2.78	3	4.29	20	14.49	7	4.70	27	9.41
women	6	12.00	0	0.00	0	0.00	0	0.00	3	16.67	1	2.33	8	22.22	4	5.71	9	6.52	13	8.72	22	7.67
older adults	1	2.00	0	0.00	0	0.00	12	26.09	0	0.00	0	0.00	0	0.00	2	2.86	13	9.42	2	1.34	15	5.23
farmers	7	14.00	2	15.38	0	0.00	0	0.00	1	5.56	0	0.00	3	8.33	0	0.00	10	7.25	3	2.01	13	4.53
low income populations	1	2.00	1	7.69	0	0.00	1	2.17	0	0.00	1	2.33	1	2.78	5	7.14	3	2.17	7	4.70	10	3.48
people experiencing homelessness/ living in the streets	2	4.00	0	0.00	0	0.00	3	6.52	1	5.56	2	4.65	1	2.78	0	0.00	6	4.35	3	2.01	9	3.14
people with underlying medical conditions	0	0.00	0	0.00	1	9.09	5	10.87	1	5.56	1	2.33	0	0.00	1	1.43	7	5.07	2	1.34	9	3.14
people living with disabilities	1	2.00	0	0.00	0	0.00	6	13.04	0	0.00	1	2.33	0	0.00	1	1.43	7	5.07	2	1.34	9	3.14
rural/remote areas	1	2.00	1	7.69	0	0.00	0	0.00	0	0.00	0	0.00	1	2.78	3	4.29	2	1.45	4	2.68	6	2.09
informal sector workers/daily wage earners	1	2.00	2	15.38	0	0.00	0	0.00	1	5.56	0	0.00	0	0.00	2	2.86	4	2.90	2	1.34	6	2.09
frontline workers – healthcare professionals	1	2.00	0	0.00	0	0.00	3	6.52	0	0.00	0	0.00	0	0.00	0	0.00	4	2.90	0	0.00	4	1.39
frontline workers - volunteers	0	0.00	0	0.00	0	0.00	2	4.35	0	0.00	0	0.00	0	0.00	2	2.86	2	1.45	2	1.34	4	1.39
Indigenous Peoples	2	4.00	1	7.69	0	0.00	0	0.00	0	0.00	0	0.00	0	0.00	0	0.00	3	2.17	0	0.00	3	1.05
other	1	2.00	2	15.38	3	27.27	9	19.57	0	0.00	3	6.98	2	5.56	10	14.29	15	10.87	15	10.07	30	10.45
undefined vulnerable population	1	2.00	0	0.00	2	18.18	8	17.39	1	5.56	2	4.65	2	5.56	8	11.43	12	8.70	12	8.05	24	8.36
undefined	2	4.00	1	7.69	0	0.00	3	6.52	1	5.56	0	0.00	5	13.89	4	5.71	7	5.07	9	6.04	16	5.57

ASPECT OF COVID-19 ADDRESSED BY INTERVENTION [a]																				
quarantine measures	35	70.00	9	69.23	5	45.45	18	39.13	11	61.11	8	18.60	16	44.44	16	22.86	78	56.52	40	26.85	118	41.11
unemployment/reduced cash flow	24	48.00	3	23.08	0	0.00	3	6.52	6	33.33	11	25.58	6	16.67	15	21.43	36	26.09	32	21.48	68	23.69
school feeding program closures	8	16.00	0	0.00	0	0.00	1	2.17	4	22.22	0	0.00	7	19.44	22	31.43	13	9.42	29	19.46	42	14.63
disrupted supply chain	4	8.00	5	38.46	0	0.00	0	0.00	1	5.56	3	6.98	6	16.67	7	10.00	10	7.25	16	10.74	26	9.06
environmental vulnerabilities	8	16.00	4	30.77	0	0.00	2	4.35	1	5.56	0	0.00	3	8.33	7	10.00	15	10.87	10	6.71	25	8.71
increased cost of goods	2	4.00	1	7.69	1	9.09	1	2.17	1	5.56	8	18.60	1	2.78	6	8.57	6	4.35	15	10.07	21	7.32
displacement/conflict	1	2.00	2	15.38	0	0.00	0	0.00	1	5.56	2	4.65	2	5.56	3	4.29	4	2.90	7	4.70	11	3.83
existing food insecurity/malnutrition	1	2.00	0	0.00	0	0.00	1	2.17	4	22.22	0	0.00	3	8.33	0	0.00	6	4.35	3	2.01	9	3.14
other	6	12.00	0	0.00	0	0.00	1	2.17	1	5.56	3	6.98	2	5.56	8	11.43	8	5.80	13	8.72	21	7.32
undefined	3	6.00	0	0.00	6	54.55	24	52.17	2	11.11	22	51.16	7	19.44	9	12.86	35	25.36	38	25.50	73	25.44
PILLARS OF FOOD SECURITY																				
**availability**[b]	40	80.00	11	84.62	11	100.00	43	93.48	14	77.78	24	55.81	11	30.56	50	71.43	119	86.23	85	57.05	204	71.08
distribution	32	80.00	10	90.91	11	100.00	43	100.00	14	100.00	21	87.50	9	81.82	48	96.00	110	92.44	78	91.76	188	92.16
production	9	28.13	1	9.09	0	0.00	1	2.33	1	7.14	3	12.50	3	27.27	4	8.00	12	10.08	10	11.76	22	10.78
exchange	0	0.00	0	0.00	0	0.00	0	0.00	1	7.14	1	4.17	1	9.09	5	10.00	1	0.84	7	8.24	8	3.92
**access**[b]	13	26.00	3	23.08	1	9.09	8	17.39	6	33.33	23	53.49	6	16.67	23	32.86	31	22.46	52	34.90	83	28.92
affordability	12	92.31	2	66.67	1	100.00	6	75.00	6	100.00	21	91.30	3	50.00	22	95.65	27	87.10	46	88.46	73	87.95
allocation	1	7.69	1	33.33	0	0.00	2	25.00	0	0.00	2	8.70	3	50.00	1	4.35	4	12.90	6	11.54	10	13.70
preference	0	0.00	0	0.00	0	0.00	0	0.00	0	0.00	1	4.35	0	0.00	0	0.00	0	0.00	1	1.92	1	10.00
**utilization**[b]	13	26.00	0	0.00	1	9.09	3	6.52	1	5.56	3	6.98	21	58.33	14	20.00	18	13.04	38	25.50	56	19.51
nutritional value	11	84.62	0	0.00	0	0.00	0	0.00	1	100.00	2	66.67	20	95.24	10	71.43	12	66.67	32	84.21	44	78.57
preparation and consumption	1	7.69	0	0.00	1	100.00	3	100.00	0	0.00	1	33.33	1	4.76	1	7.14	5	27.78	3	7.89	8	14.29
food safety	0	0.00	0	0.00	0	0.00	0	0.00	0	0.00	0	0.00	1	4.76	2	14.29	0	0.00	3	7.89	3	5.36
cultural acceptability	1	7.69	0	0.00	0	0.00	1	33.33	0	0.00	0	0.00	0	0.00	1	7.14	2	11.11	1	2.63	3	5.36
health status	1	7.69	0	0.00	0	0.00	0	0.00	0	0.00	0	0.00	1	4.76	0	0.00	1	5.56	1	2.63	2	3.57
**stability**[b]	5	10.00	4	30.77	0	0.00	0	0.00	3	16.67	3	6.98	2	5.56	10	14.29	12	8.70	15	10.07	27	9.41

stable supply	2	40.00	2	50.00	0	0.00	0	0.00	3	100.00	3	100.00	1	50.00	7	70.00	7	58.33	11	73.33	18	66.67
environmental stability	3	60.00	2	50.00	0	0.00	0	0.00	0	0.00	0	0.00	1	50.00	3	30.00	5	41.67	4	26.67	9	33.33
PARTNERS[a]																				
national government	13	26.00	0	0.00	4	36.36	18	39.13	0	0.00	10	23.26	26	72.22	34	48.57	35	25.36	70	46.98	105	36.59
other NGO	14	28.00	4	30.77	6	54.55	10	21.74	4	22.22	16	37.21	5	13.89	9	12.86	38	27.54	30	20.13	68	23.69
community volunteers	10	20.00	1	7.69	3	27.27	26	56.52	0	0.00	7	16.28	1	2.78	1	1.43	40	28.99	9	6.04	49	17.07
UN agency	5	10.00	0	0.00	0	0.00	4	8.70	1	5.56	14	32.56	6	16.67	12	17.14	10	7.25	32	21.48	42	14.63
business	5	10.00	2	15.38	0	0.00	14	30.43	4	22.22	7	16.28	0	0.00	3	4.29	25	18.12	10	6.71	35	12.20
local government	15	30.00	2	15.38	0	0.00	1	2.17	0	0.00	3	6.98	1	2.78	2	2.86	18	13.04	6	4.03	24	8.36
health workers	5	10.00	0	0.00	1	9.09	5	10.87	0	0.00	1	2.33	6	16.67	0	0.00	11	7.97	7	4.70	18	6.27
regional government	2	4.00	0	0.00	0	0.00	2	4.35	1	5.56	0	0.00	4	11.11	2	2.86	5	3.62	6	4.03	11	3.83
faith-based organization	5	10.00	0	0.00	0	0.00	0	0.00	0	0.00	0	0.00	1	2.78	0	0.00	5	3.62	1	0.67	6	2.09
other	7	14.00	2	15.38	0	0.00	1	2.17	2	11.11	7	16.28	7	19.44	0	0.00	12	8.70	14	9.40	26	9.06
undefined	11	22.00	7	53.85	2	18.18	5	10.87	8	44.44	12	27.91	2	5.56	23	32.86	33	23.91	37	24.83	70	24.39

[a] The total interventions will not equal to n=287 because the category was not mutually exclusive and could have incorporated more than one component.
[b] Within each pillar of food security (i.e. availability, access, utilization, and stability) the various components comprising the pillar were calculated as proportions of the number of interventions within the corresponding pillar.

ICRC=International Committee of the Red Cross; IFRC=International Federation of Red Cross; CARE=Cooperative for Assistance and Relief Everywhere; UNHCR=United Nations Refugee Agency; UNICEF=United Nations Children's Fund; WFP=World Food Programme; INGO=International non-governmental organization; UN=United Nations
